# GEO-Based VLBI for Deep Space Navigation: Time-Position Error Modeling and Performance Analysis

**DOI:** 10.3390/s26216707

**Published:** 2026-10-23

**Authors:** Moshe Golani, Yoram Rozen, Hector Rotstein

**Affiliations:** 1 Department of Physics, Technion—Israel Institute of Technology, Haifa 3200003, Israel; rozen@physics.technion.ac.il; 2 Taub Faculty of Computer Science, Technion—Israel Institute of Technology, Haifa 3200003, Israel; hector@ee.technion.ac.il

**Keywords:** deep-space navigation, VLBI, radiometric interferometry, GEO satellites, orbit determination

## Abstract

Radiometric interferometry, implemented through ground-based Very Long Baseline Interferometry (VLBI) and Delta Differential One-Way Ranging (Delta-DOR), is a powerful technique for angular spacecraft navigation. The previously proposed concept of Radiometric Interferometry Navigation using Geostationary Earth Orbit (GEO) Satellites (RINGS) replaces terrestrial stations with GEO satellites, so that its basic measurement unit is a single GEO-GEO interferometric baseline. This paper analyzes the two main practical error sources of this two-station sensor: residual relative clock misalignment between the satellites and uncertainty in their positions. Analytical models map both into angular error and show that the timing-induced path error is divided by the long GEO-GEO baseline. The timing chain is evaluated, including Global Navigation Satellite System (GNSS) synchronization, passive hydrogen maser (PHM) clocks, two-way inter-satellite time transfer, and relativistic corrections, and a Monte Carlo framework propagates clock and position errors through a dual-frequency angle-of-arrival estimator. For a GEO-GEO baseline of about 84,000km, residual timing errors of 10ps and 100ps contribute about 0.05nrad and 0.5nrad of angular scatter, whereas GEO position uncertainty contributes about 17nrad per meter per axis and is the dominant error driver; for the 120° three-GEO baseline these values are about 15% larger. A Sagnac-type term biases the uncorrected two-way clock estimate by about 1.25 μs and must be computed from the GEO states. Theoretical analysis supported by simulation shows that nanoradian-level angular precision is attainable with sub-meter GEO position knowledge and PHM-class timing. This is the statistical precision floor of the sensor, not its absolute accuracy after calibration.

## 1. Introduction

Very Long Baseline Interferometry (VLBI) and Delta Differential One-Way Ranging (Delta-DOR) are established radiometric techniques for deep-space navigation [1]. These methods provide highly accurate angular information by measuring the differential arrival time or phase of a spacecraft radio signal at widely separated receiving stations. In conventional deep-space navigation, the receiving stations are located on Earth, and the achievable angular accuracy depends on baseline length, signal-to-noise ratio, atmospheric calibration, station-location knowledge, clock stability, and observation geometry. These techniques continue to be refined. Recent examples are the joint processing of regenerative ranging and DOR signals, which uses the ranging clock component to resolve the integer ambiguity of the interferometric delay [2], and improved tropospheric mapping for the VLBI tracking of the Tianwen-1 Mars mission, where the receiver-side tropospheric delay remains a significant error at low elevation [3].

The RINGS concept, Radiometric Interferometry Navigation using geostationary Earth orbit (GEO) satellites, was introduced as a space-based extension of terrestrial VLBI [4]. Instead of using ground stations as the interferometric receivers, RINGS uses geostationary satellites as the receiving stations. In this sense, RINGS proposes a new type of interferometric sensor: its fundamental measurement unit is a single GEO-GEO baseline formed by two receiving satellites, in direct analogy to a two-element terrestrial VLBI array.

For the three-GEO “trio” configuration adopted in this paper, with satellites separated by approximately 120° (Section 5.4), this architecture creates a GEO-to-GEO interferometric baseline of approximately 73,000km, substantially longer than terrestrial VLBI baselines. The space-based architecture also removes receiver-side tropospheric and ionospheric path errors and improves geometric measurement availability for deep-space targets.

The first RINGS study introduced the basic measurement concept and provided a top-down stochastic estimate of the expected angle of arrival (AOA) accuracy. The present paper continues that work by analyzing the two main practical uncertainty mechanisms that dominate a GEO-based implementation: residual relative clock misalignment between the GEO receivers and uncertainty in the GEO positions. These two effects directly affect the interferometric phase observable and therefore the reconstructed angular direction to the spacecraft. Because these effects act at the level of a single interferometric baseline, the analysis in this paper characterizes the performance of the basic two-station RINGS sensor.

A further ingredient of the RINGS observable is the use of more than one carrier frequency. The phase of a single carrier is measured only modulo one cycle, so the interferometric delay is ambiguous by integer multiples of the carrier wavelength, and on a very long baseline many candidate delay values are consistent with a single-frequency measurement. Observing the same spacecraft signal at two frequencies $f_{1}$ and $f_{2}$ allows a differential (wide-lane) phase to be formed, which behaves like a phase measured at the much lower beat frequency $\Delta f=f_{2}-f_{1}$. Its effective wavelength, c/Δf, is far longer than either carrier wavelength, so the ambiguity interval is widened and the candidate solutions can be separated.

The price is a coarser phase-to-delay resolution, which is why the wide-lane solution is used to select the correct carrier-phase branch rather than as the final precision observable. Using more receiving stations (for example, the third satellite of the GEO trio) and more carrier frequencies further constrains the solution and makes the ambiguous candidates easier to distinguish. This is the standard multi-frequency principle for ambiguity reduction in long-baseline interferometry, and it is the role of the dual-frequency observable used in this paper (see Section 2.1 and Section 3.3).

This paper is an engineering error budget for the basic two-station sensor. Mission-level aspects, such as mission design, payload and equipment design, and calibration strategy, were addressed at the concept level in the first RINGS study [4] and are part of the system engineering of a dedicated platform (Section 6).

Together with the first RINGS study, which addressed the link budget, the signal-to-noise ratio and the Doppler dynamics of the GEO receivers [4], this paper completes a thorough analysis of the main error contributors of the sensor. Its contribution is threefold. First, it develops analytical models for the two dominant practical error sources, the residual clock misalignment between the GEO receivers and the uncertainty in their positions, together with the timing chain that controls the clock term: GNSS-based synchronization, onboard atomic clocks, two-way inter-satellite time transfer, relativistic corrections, and hardware-delay calibration. Second, it builds a Monte Carlo simulation model of the RINGS measurement chain, uses it to examine these error sources, and confirms the analytical results. Third, it shows that with this space-based arrangement the angular scatter of each AOA measurement can be kept within the nanoradian level achieved by terrestrial VLBI and Delta-DOR.

The AOA measurements are processed as observations in the orbit determination of the spacecraft, together with range and Doppler data, in the same way as Delta-DOR and terrestrial VLBI data [5,6]; the corresponding least-squares formulation is given in Section 2.5 for the GEO satellites. The angular scatter established here is therefore the measurement error model that RINGS contributes to the navigation solution. Combined with the system-level advantages of the space-based arrangement, namely a long baseline, no receiver-side atmosphere, and high geometric availability, these results establish RINGS as a promising complementary deep-space navigation technology, with potential for further improvement as orbit-determination and timing technologies advance.

Section 2 develops the analytical error models, Section 3 the simulation methodology, and Section 4 the Monte Carlo results. Section 5 discusses the dominant error drivers and their implications, and Section 6 concludes.

## 2. Analytical Error Modeling

This section develops the analytical error models of the two-station sensor, first for the timing chain (Section 2.1, Section 2.2, Section 2.3 and Section 2.4) and then for the GEO positions and the hardware delays (Section 2.5 and Section 2.6).

### 2.1. Clock Error Mapping and Synchronization Requirement

Precise relative timing between the GEO receivers is essential for RINGS. The interferometric observable is the difference between the propagation paths of the spacecraft signal to the two GEO receivers, measured through the phase difference. In this subsection, δt denotes the residual relative clock offset, also called clock misalignment, between the two receivers in a given measurement, and $\sigma_{t}$ denotes its standard deviation over the ensemble of measurements. A clock offset δt cannot be distinguished from a path difference cδt, so the standard deviation of the resulting path-length error is

(1) $$\sigma_{L}=c\sigma_{t}$$

where *c* is the speed of light. For a distant source, the path difference *L* is the projection of the GEO-GEO baseline on the line of sight,

(2) L=Bcosθ

where *B* is the GEO-GEO baseline length and θ is the angle between the baseline and the line of sight to the spacecraft. Linearizing Equation (2), a small angle change δθ corresponds to the path-difference change

(3) δL=−Bsinθδθ

A clock offset δt adds to the measured path difference the error

(4) δL=cδt

which the estimator cannot separate from a geometric change. Equating Equations (3) and (4) gives the angular error caused by the clock offset,

(5) $$\delta \theta_{\mathrm{clk}}\approx -\frac{c\delta t}{B\sin\theta }$$

Here δL is the error of one measurement and $\sigma_{L}$ of Equation (1) is its standard deviation. Thus, the clock-induced angular error is linear in the residual clock misalignment δt, with a sensitivity set by c/(Bsinθ). This is a small-signal perturbation result: sinθ is bounded within (0,1] and fixed by the instantaneous baseline-to-line-of-sight geometry at the time of observation, so it mainly rescales the sensitivity by a consistent, order-unity geometric factor. Away from the singular case sinθ→0, the standard deviation of the clock-induced angular error used for error-budget sizing is

(6) $$\sigma_{\theta ,\mathrm{clk}}\approx \frac{\sigma_{L}}{B}=\frac{c\sigma_{t}}{B}$$

Equation (6) is the key clock-sizing relation for RINGS. A clock misalignment produces the same path-length error $\sigma_{L}=c\sigma_{t}$ regardless of the interferometer architecture, but the resulting angular error is divided by the interferometric baseline. Since the GEO-GEO baseline is much longer than a terrestrial VLBI baseline, the same residual timing error produces a smaller angular perturbation. For two systems with the same timing residual, the relative clock-induced angular error scales as

(7) $$\frac{\sigma_{\theta ,\mathrm{RINGS}}}{\sigma_{\theta ,\mathrm{terr}}}\approx \frac{B_{\mathrm{terr}}}{B_{\mathrm{RINGS}}}$$

In nanoradians, Equation (6) becomes

(8) $$\sigma_{\theta ,\mathrm{clk}}\left[\mathrm{nrad}\right]\approx 299.8\frac{\sigma_{t}\left[\mathrm{ps}\right]}{B[\mathrm{km}]}$$

In the rest of this paper the baseline is that of the trio configuration. With $r_{\mathrm{GEO}}\approx 42,164\;\mathrm{km}$, the corresponding GEO-GEO chord length is

(9) $$B_{120}=2r_{\mathrm{GEO}}\sin\left(\frac{120{}^{\circ}}{2}\right)\approx 73,030\;\mathrm{km}$$

and this value, rounded to B≈73,000km in the numerical estimates that follow, is used as the representative RINGS baseline throughout this paper. For comparison, the near-antipodal, two-satellite configuration originally considered in the first RINGS study [4] approaches the maximum possible GEO chord length,

(10) $$B_{\max}\approx 2r_{\mathrm{GEO}}\approx 84,328\;\mathrm{km}$$

The trio baseline is 13.4% shorter than this bound; the reasons for accepting this are given in Section 5.4.

The first RINGS study estimated terrestrial-VLBI-order angular performance at the few-nanoradian level, with a representative value of approximately 4nrad [4]. If the full clock contribution were allowed to reach this value, Equation (6) would give, for the trio baseline,

(11) $$\sigma_{t}\approx 0.97\;\mathrm{ns}\;\left(\sigma_{\theta }=4\;\mathrm{nrad}\right)$$

However, the clock contribution should be only a fraction of the total angular error budget because GEO position uncertainty, receiver noise, calibration residuals, and phase ambiguity also contribute. A more appropriate clock-allocation reference is therefore 1nrad, which corresponds to

(12) $$\sigma_{t}\approx 244\;\mathrm{ps}\;\left(\sigma_{\theta }=1\;\mathrm{nrad}\right)$$

Equation (12) provides the basic timing boundary: residual relative clock error must be kept below the few-hundred-picosecond level to keep the clock contribution below 1nrad. In practice, a more conservative design target preserves margin:

(13) $$\sigma_{t}\approx 30\;\mathrm{ps}$$

For the trio baseline, this corresponds to an angular contribution of approximately

(14) $$\sigma_{\theta }=\frac{c\sigma_{t}}{B}\approx 0.12\;\mathrm{nrad}$$

Thus, clock synchronization at the level of a few tens of picoseconds keeps the clock-induced stochastic angular contribution well below the nanoradian level for the representative RINGS trio baseline.

Global Navigation Satellite System (GNSS) time can anchor absolute time for spacecraft at GEO altitude when signal availability, receiver sensitivity and tracking geometry are sufficient [7]. The question is whether GNSS-based synchronization alone could also meet the relative timing requirement.

Public GPS performance specifications report time-transfer accuracy relative to UTC (USNO), the United States Naval Observatory realization of Coordinated Universal Time, at the tens-of-nanoseconds level [8]. Dedicated GNSS time-transfer methods with high-quality equipment and post-processing can provide improved timing performance under favorable conditions [9]. Therefore, this study uses

(15) $$\sigma_{\mathrm{GNSS}}=1\;\mathrm{ns}$$

as an optimistic one-sigma GNSS-based timing uncertainty, a favorable reference case rather than a guaranteed GEO performance level.

For two GEO satellites independently synchronized to GNSS time, the relative timing uncertainty is approximately

(16) $$\sigma_{t}=\sqrt{2}\sigma_{\mathrm{GNSS}}\approx 1.4\;\mathrm{ns}$$

For the trio baseline, this maps into an angular uncertainty of approximately

(17) $$\sigma_{\theta }=\frac{c\sigma_{t}}{B}\approx 5.8\;\mathrm{nrad}$$

This value is within the terrestrial VLBI order of magnitude, but too large for the clock contribution to remain a small fraction of the RINGS error budget. GNSS can therefore anchor absolute time, but the relative timing requires a dedicated onboard frequency reference and direct inter-satellite synchronization.

The above estimates assume that phase ambiguity is resolved and that the estimator remains on the correct phase branch, which is the role of the dual-frequency observable introduced in Section 1 and formulated in Section 3.3. Because a residual clock offset enters as a delay, the mapping of Equation (8) does not depend on whether the delay is extracted from a single carrier or from the wide-lane combination: the second frequency changes the ambiguity interval, not the delay-to-angle mapping.

### 2.2. Clock Stability over the Coherent Integration Window

The short-term stability of an onboard frequency reference is commonly described by the Allan deviation $\sigma_{y}\left(\tau \right)$, where τ is the averaging time [10,11,12]. It is a measure of fractional frequency stability, not of clock time offset: it quantifies fractional frequency fluctuations, which accumulate into timing error over a finite observation interval.

For RINGS, the relevant time interval is the coherent VLBI integration window at the two GEO receiving stations. In this study, the representative coherent integration window, which is also the observation arc of the simulations in Section 3 and Section 4, is taken as

(18) τ=300s

The onboard clock must therefore keep its time variation over this interval below the required level. Passive hydrogen masers (PHMs) are a natural candidate for this requirement because they combine excellent short-term frequency stability, low long-term frequency drift, and demonstrated space qualification.

PHM clocks have already been developed, qualified, and flown in European satellite-navigation missions, including Galileo [13]. As a representative onboard clock technology for RINGS, this study considers a space-qualified PHM of the Mini-PHM class. The Mini-PHM is used here as a reference performance level, not as a final payload selection. Representative Allan deviation values reported for the Leonardo space PHM and Mini-PHM class are summarized in Table 1 [14].

The coherent integration window used in the RINGS simulation, 300s, is between the tabulated 100s and 1000s values. Assuming white frequency-modulation noise over this interval, the Allan deviation scales approximately as

(19) $$\sigma_{y}\left(\tau \right)\propto \tau^{-1/2}$$

Reducing the averaging time from 1000s to 300s therefore increases the Allan deviation, as expected in the white-frequency-noise regime, where stability improves with longer averaging. Using the 1000s reference value from Table 1, the corresponding estimate is

(20) $$\sigma_{y}\left(300\;s\right)=\sigma_{y}\left(1000\;s\right)\sqrt{\frac{1000}{300}}\approx 4.0\times 10^{-14}$$

This value is also consistent, at order-of-magnitude level, with interpolation between the 100s and 1000s Allan deviation values in Table 1.

To estimate the timing impact during the coherent integration window, the fractional frequency stability is converted into an RMS time deviation. For white frequency-modulation noise, an approximate relation is

(21) $$\sigma_{x}\left(\tau \right)\approx \frac{\tau \sigma_{y}\left(\tau \right)}{\sqrt{3}}$$

where $\sigma_{x}\left(\tau \right)$ represents the short-term stochastic time deviation accumulated over the interval τ.

Substitution of τ=300s gives

(22) $$\sigma_{x}\left(300\;s\right)=\frac{300\cdot 4.0\times 10^{-14}}{\sqrt{3}}\approx 6.9\times 10^{-12}\;s\approx 7\;\mathrm{ps}$$

For two independent GEO satellite clocks, the relative timing uncertainty between the two receiving stations is approximately

(23) $$\sigma_{x,\mathrm{baseline}}=\sqrt{2}\sigma_{x}\approx 10\;\mathrm{ps}$$

This value is the short-term stochastic timing instability over the 300s integration window. It does not include slowly varying clock offset, temperature sensitivity, aging, or calibration residuals, which are removed by calibration and two-way inter-satellite time transfer. This is feasible because Mini-PHM-class clocks have a long-term frequency drift below $3\times 10^{-15}$ per day and a temperature sensitivity below $2\times 10^{-14}/{}^{\circ}C$ [14], so these terms vary on timescales much longer than the few-minute integration window.

### 2.3. Two-Way Synchronization and Subsequent Clock Residuals

Clock stability (Equation (23)) does not determine the clock offset itself, its slow drift, or the internal hardware delays, which must be calibrated. RINGS therefore uses a two-way inter-satellite synchronization concept analogous to classical Two-Way Satellite Time and Frequency Transfer (TWSTFT) [15,16,17,18]. The method is adapted here to a direct GEO-GEO link.

The two-way link requires direct inter-satellite line of sight, which the trio geometry of Figure 1 provides (Section 5.4).

The two-way synchronization and ranging concept is illustrated in Figure 2.

Let $T_{1}$ be the transmission time from satellite 1 and $T_{1,2}$ the corresponding reception time at satellite 2. Similarly, let $T_{2}$ and $T_{2,1}$ denote the reverse transmission and reception times. The timestamp equations are

(24) $$\begin{array}{cc} T_{1,2}\; & =T_{1}+\tau +\Delta +p_{2} \end{array}$$

(25) $$\begin{array}{cc} T_{2,1}\; & =T_{2}+\tau -\Delta +p_{1} \end{array}$$

where τ is the one-way light time between the satellites, Δ is the clock offset of satellite 2 relative to satellite 1, and $p_{1},p_{2}$ are internal hardware and processing delays.

The two-way clock-offset estimator is

(26) $$\hat{\Delta }=\frac{\left(T_{1,2}-T_{1}\right)-\left(T_{2,1}-T_{2}\right)+\Delta p_{\mathrm{cal}}}{2}$$

where $\Delta p_{\mathrm{cal}}$ is the calibrated value of the differential internal delay $p_{1}-p_{2}$ between the two GEO satellites. The residual clock offset δt of Section 2.1 is the error of this estimate, $\delta t=\hat{\Delta }-\Delta$, after synchronization. Equation (26) assumes reciprocal propagation, that is, the same light time τ on both legs, so that τ cancels. Departures from this assumption between moving GEO satellites are analyzed with the relativistic effects in Section 2.4, where the corrected estimator is given.

The sum of the two timestamp equations preserves the geometric light-time information:

(27) $$\left(T_{1,2}-T_{1}\right)+\left(T_{2,1}-T_{2}\right)=2\tau +\left(p_{1}+p_{2}\right)$$

Defining the calibrated sum of internal delays as

(28) $$p_{\Sigma ,\mathrm{cal}}=p_{1}+p_{2}$$

the one-way light time can be estimated as

(29) $$\hat{\tau }=\frac{\left(T_{1,2}-T_{1}\right)+\left(T_{2,1}-T_{2}\right)-p_{\Sigma ,\mathrm{cal}}}{2}$$

In free space,

(30) $$\tau =\frac{B}{c}$$

and therefore the inter-satellite range can be estimated as

(31) $$\hat{B}=c\hat{\tau }$$

This provides an important additional benefit for RINGS. The same inter-satellite link used for two-way time transfer can also contribute to GEO-GEO baseline estimation. If the GEO satellites are equipped with a dedicated laser ranging link, the along-baseline component of the baseline can be further constrained and calibrated independently of the radio-frequency (RF) timing channel. Direct inter-satellite communication thus provides both relative clock synchronization and a navigation aid for the baseline knowledge required by the interferometric measurement.

The residual clock contribution after synchronization can now be evaluated using Equation (8). For the 120° three-GEO trio baseline in Equation (9), the PHM-class short-term stochastic timing level from Equation (23) gives

(32) $$\sigma_{\theta ,\mathrm{clk}}\approx 299.8\frac{10}{73,030}=0.041\;\mathrm{nrad}$$

A 100ps residual timing case, representing a conservative residual after imperfect correction of slowly varying or deterministic propagation effects, gives

(33) $$\sigma_{\theta ,\mathrm{clk}}\approx 299.8\frac{100}{73,030}=0.41\;\mathrm{nrad}$$

Representative values are summarized in Table 2, which compares a representative terrestrial VLBI-scale baseline with the RINGS trio baseline.

The same 100ps residual gives 3.75nrad on an 8000km terrestrial baseline but only 0.41nrad on the RINGS trio baseline, so after PHM-class timing and two-way synchronization the residual stochastic clock contribution remains well below the nanoradian level.

### 2.4. Relativistic and Moving-Endpoint Timing Corrections

At picosecond-level timing accuracy, the GEO-GEO time-transfer model must account for three distinct effects: (i) the differential relativistic clock rate between the two satellites, (ii) the Shapiro propagation delay in the Earth gravitational field, and (iii) the Sagnac-type non-reciprocal light-time asymmetry caused by the motion of the two endpoints during signal propagation. An onboard atomic clock provides a stable proper-time reference, but it does not correct any of these effects by itself. They must be handled in the time-tag reduction, the two-way light-time solution, and the baseline-estimation model, as in other high-precision microwave and optical time-transfer links [19,20,21]. The three effects differ strongly in size and, more importantly, in whether they cancel in a two-way exchange: effects (i) and (ii) are reciprocal or negligible, whereas effect (iii) is not reciprocal and therefore does not cancel.

For the representative RINGS trio baseline of B≈73,000km, the GEO-GEO light time is [6]

(34) $$\tau_{\mathrm{GEO}-\mathrm{GEO}}=\frac{B}{c}\approx 0.24\;s$$

During this interval each GEO satellite moves by about $0.24\;s\times 3.07\;\mathrm{km}\;s^{-1}\approx 0.7\;\mathrm{km}$, and the signal crosses a region of the Earth gravitational field in which the coordinate speed of light is measurably below *c*.

Effect (i): differential relativistic clock rate.

To first order in $1/c^{2}$, the relation between the proper time $\tau_{i}^{p}$ of satellite *i* and a common reference coordinate time $t_{R}$ is [19,22] (the superscript p distinguishes proper time from the light times τ used elsewhere)

(35) $$\frac{d\tau_{i}^{p}}{dt_{R}}=1-\frac{U_{i}}{c^{2}}-\frac{v_{i}^{2}}{2c^{2}}$$

where $U_{i}=\mu_{⊕}/r_{i}$ is the (positive) gravitational potential of a point-mass Earth at satellite *i*, $r_{i}$ is its geocentric distance, $\mu_{⊕}=GM_{⊕}\approx 3.986\times 10^{14}\;m^{3}\;s^{-2}$ is the Earth gravitational parameter (the product of the gravitational constant *G* and the Earth mass $M_{⊕}$) [23], and $v_{i}$ is the inertial speed of satellite *i*. For two GEO receivers, Equation (35) gives the approximate differential rate [22]

(36) $$\delta {\dot{\tau }}_{\mathrm{rel}}^{p}\approx -\frac{\delta U}{c^{2}}-\frac{v_{\mathrm{GEO}}\delta v}{c^{2}}$$

where $\delta U=U_{2}-U_{1}$, $v_{\mathrm{GEO}}$ is the nominal GEO speed, and δv is the differential speed error between the two satellites. For two nominal GEO satellites in the same orbital shell, the gravitational and kinematic terms are common-mode. For a circular orbit of radius *r*, the orbital speed satisfies $v^{2}=\mu_{⊕}/r$ [22], so that $U+v^{2}/2=3\mu_{⊕}/\left(2r\right)$ and Equation (35) reduces to $d\tau^{p}/dt_{R}=1-3\mu_{⊕}/\left(2c^{2}r\right)$. A small radial mismatch δr between the two orbit radii then changes the rate of one clock relative to the other by the derivative of this expression with respect to *r*, multiplied by δr:

(37) $$\begin{array}{cc} {\dot{\tau }}^{p}\left(r\right)\; & =1-\frac{3\mu_{⊕}}{2c^{2}r},\\ \left|\delta {\dot{\tau }}_{\mathrm{rel}}^{p}\right|\; & =\left|\frac{d{\dot{\tau }}^{p}}{dr}\right|\delta r\approx \frac{3\mu_{⊕}}{2c^{2}r_{\mathrm{GEO}}^{2}}\delta r \end{array}$$

where *c* is the speed of light, $\mu_{⊕}$ is the Earth gravitational parameter defined above, $r_{\mathrm{GEO}}$ is the GEO orbit radius, and δr is the radial mismatch between the two orbits.

Using $r_{\mathrm{GEO}}\approx 42,164\;\mathrm{km}$, Equation (37) gives

(38) $$\left|\delta {\dot{\tau }}_{\mathrm{rel}}^{p}\right|\approx 3.7\times 10^{-18}\left(\frac{\delta r}{1\;m}\right)$$

Over the 300s integration window, Equation (38) gives

(39) $$\delta \tau_{\mathrm{rel}}^{p}\approx 1.1\times 10^{-15}\left(\frac{\delta r}{1\;m}\right)\;s$$

Thus, even a 10m radial mismatch gives only about 0.01ps over the RINGS integration window, and effect (i) is negligible compared with the few-picosecond to tens-of-picoseconds timing levels considered in this work.

Effect (ii): Shapiro propagation delay.

The signal travels through the Earth gravitational field, where the coordinate speed of light is slightly below *c*. The dominant first-order excess delay for a link between two points at geocentric distances $r_{1}$ and $r_{2}$ separated by *B* is the Shapiro delay [22,24,25]

(40) $$\Delta t_{\mathrm{Shapiro}}\approx \frac{2\mu_{⊕}}{c^{3}}\ln\left(\frac{r_{1}+r_{2}+B}{r_{1}+r_{2}-B}\right)$$

where $\mu_{⊕}$ is defined above. For $r_{1}\approx r_{2}\approx r_{\mathrm{GEO}}$ and B≈73,000km, Equation (40) gives $\Delta t_{\mathrm{Shapiro}}\approx 78\;\mathrm{ps}$, equivalent to about 2.3cm of additional path. The delay is the same for the forward and reverse directions to the order considered here, so it is a reciprocal term that cancels in the two-way combination. It remains, however, in the one-way light time used to convert the two-way observable to a range and a baseline. It is deterministic and, if ignored, would bias the inter-satellite range by about 2.3cm. It is therefore computed from Equation (40) and removed in the baseline-estimation process [6].

Effect (iii): moving-endpoint (Sagnac-type) asymmetry.

Although the instantaneous GEO-GEO distance is the same in both directions, the forward and reverse signals connect different transmission and reception events, and the two satellites move during the light time in Equation (34). To first order in v/c, the coordinate light times between satellite 1 and 2, with unit vector ${\hat{n}}_{12}$ from 1 to 2, are [6,26]

$$\tau_{12}\approx \frac{B}{c}+\frac{B}{c^{2}}\;{\hat{n}}_{12}\;\cdot v_{2},\;\tau_{21}\approx \frac{B}{c}-\frac{B}{c^{2}}\;{\hat{n}}_{12}\;\cdot v_{1}$$

where $v_{1}$ and $v_{2}$ are the inertial velocities, and the receiver motion lengthens the path when it recedes from the transmitter and shortens it when it approaches. The non-reciprocal part is

(41) $$\Delta \tau_{\mathrm{nr}}=\tau_{12}-\tau_{21}\approx \frac{B}{c^{2}}\;{\hat{n}}_{12}\;\cdot \;\left(v_{1}+v_{2}\right)$$

For two satellites on a circular GEO orbit with angular rate $\omega_{\mathrm{GEO}}$ and angular separation α, Equation (41) reduces to the Sagnac form [22,26]

(42) $$\Delta \tau_{\mathrm{nr}}=\frac{2\omega_{\mathrm{GEO}}r_{\mathrm{GEO}}^{2}}{c^{2}}\sin\alpha =\frac{4\;\omega_{\mathrm{GEO}}A}{c^{2}},\;A=\frac{1}{2}r_{\mathrm{GEO}}^{2}\sin\alpha$$

where *A* is the area of the triangle formed by the two satellites and the Earth center. For the 120° trio separation, $\Delta \tau_{\mathrm{nr}}\approx 2.5\;\mu s$. This value is independent of the reference frame: in an Earth-fixed frame the GEO endpoints are nearly stationary, and the same term appears as the Sagnac effect of the rotating frame. Table 3 gives the size of the term for representative GEO-GEO separations, together with the Shapiro delay of Equation (40) for comparison. Figure 3 shows the three effects of this subsection over the full range of separations. The asymmetry is larger than the Shapiro delay by about four orders of magnitude for all separations in the table, which is why it dominates the raw two-way observable.

The consequence for two-way time transfer is the following. Write the one-way coordinate light times as $\tau_{12}=\bar{\tau }+X$ and $\tau_{21}=\bar{\tau }-X$, with $\bar{\tau }=B/c+\Delta t_{\mathrm{Shapiro}}$ and $X=\Delta \tau_{\mathrm{nr}}/2\approx 1.25\;\mu s$. The leg that propagates along the direction of orbital motion is lengthened by *X* and the opposite leg is shortened by *X*, so the sum $\tau_{12}+\tau_{21}=2\bar{\tau }$ (the turn-around time) is unchanged to first order. The round-trip delay therefore does not reveal the effect, while the difference of the two one-way readings, which is the quantity that carries the clock offset, is shifted by 2X. An uncorrected estimator therefore returns a clock offset that is biased by X≈1.25μs, many orders of magnitude above the picosecond target. The residual second-order term in the round trip is only of order 10ps (about −6ps for the trio) and is captured by solving the light-time equations with the satellite states.

The corrected two-way clock-offset estimator, in the form used for TWSTFT [15,16,17,18], is therefore

(43) $$\hat{\Delta }=\frac{\left(T_{1,2}-T_{1}\right)-\left(T_{2,1}-T_{2}\right)-\Delta \tau_{\mathrm{nr}}+\Delta p_{\mathrm{cal}}}{2}$$

where $\Delta p_{\mathrm{cal}}$ is the calibrated differential internal hardware delay. Equation (43) shows that two-way synchronization removes the common reciprocal propagation delay, but the non-reciprocal moving-endpoint term and the hardware-delay term must still be modeled or calibrated.

Sensitivity and architecture implication.

The correction $\Delta \tau_{\mathrm{nr}}$ is deterministic and is computed from the GEO states through Equation (41). Its accuracy is set by the knowledge of the inertial velocities of the two satellites, through the component of $v_{1}+v_{2}$ along ${\hat{n}}_{12}$, with sensitivity $B/(2c^{2})\approx 0.41\;\mathrm{ns}$ per $m\;s^{-1}$. A 1ps contribution therefore requires this velocity component to be known to about $2.5\;\mathrm{mm}\;s^{-1}$, whereas a 1m position error contributes only about 0.05ps and is negligible. Because the time stamps recorded at each satellite are shifted by the effect, the correction must be applied in the time-tag reduction, either onboard each satellite using its own state and the partner state exchanged over the link, or in ground post-processing. The GEO architecture must therefore provide velocity knowledge at the $\mathrm{mm}\;s^{-1}$ level for every satellite in the constellation.

Summary.

Effect (i) is negligible, effect (ii) cancels in the clock offset but is removed from the range, and effect (iii), the dominant one, is computed from the GEO states. After these corrections, the remaining timing error is governed mainly by the knowledge of the GEO position and velocity of each satellite (Section 2.5), which sets the baseline error, and by the calibration of the internal hardware delays (Section 2.6), rather than by an independent stochastic relativistic clock error.

### 2.5. GEO Position Knowledge and Baseline Error Representation

In a practical RINGS implementation, GEO orbit determination (OD) would have to be performed at the highest available precision level, using high-fidelity force modeling, high-quality tracking data, direct inter-satellite measurements, and advanced estimation and filtering methods [5,27]. The full development of such a dedicated GEO OD solution is outside the scope of this paper. Instead, this study uses representative residual GEO position uncertainty levels after OD and calibration, and propagates them through the RINGS interferometric measurement model.

The geometric quantity that enters the RINGS interferometric phase is the GEO-GEO baseline vector during the observation arc. The baseline is

(44) $$B\left(t\right)=r_{2}\left(t\right)-r_{1}\left(t\right)$$

where $r_{1}\left(t\right)$ and $r_{2}\left(t\right)$ are the position vectors of the two GEO receiving satellites. Residual errors in the GEO position solutions perturb the estimated baseline. Therefore,

(45) $$\hat{B}\left(t\right)=B\left(t\right)+\delta B\left(t\right)$$

with

(46) $$\delta B\left(t\right)=\delta r_{2}\left(t\right)-\delta r_{1}\left(t\right)$$

Thus, the interferometric phase is governed by the full three-dimensional baseline vector, that is, by the position of each of the two satellites. An error that is common to both satellites cancels in the difference, but the independent part of each satellite position error does not. Inter-satellite ranging constrains only the component of δB along the line joining the satellites. It does not constrain the transverse components, which still map into the projected baseline and hence into the angle. A precise baseline length therefore does not by itself guarantee an accurate baseline direction, and the orbit determination of each satellite remains the primary requirement.

Equation (46) is the exact signed difference of the two position-error vectors, which is the quantity that enters the interferometric phase. The subtraction does not by itself reduce the error. For a worst-case bound, the triangle inequality gives $|\delta B|\leq |\delta r_{2}|+|\delta r_{1}|$, that is, the moduli add. For random errors, the appropriate description is the covariance of the difference (Equation (50)): independent errors add in variance, and only errors that are positively correlated between the two satellites, such as common force-model or reference-frame errors, partially cancel in the baseline. The simulations of Section 3 assume independent position errors for the two satellites and therefore take no credit for such cancellation.

Using standard statistical orbit-determination notation [5], the linearized measurement model around an a priori trajectory can be written as

(47) y≃AΔx+ϵ

where y is the measurement residual vector, Δx is the state correction, A is the measurement sensitivity matrix, and ϵ is the measurement noise.

For a standard weighted least-squares or batch-estimation formulation [5], the formal state covariance is

(48) $$P={\left(A^{T}WA+P_{0}^{-1}\right)}^{-1}$$

where W is the measurement weighting matrix and $P_{0}$ is the a priori covariance matrix. The term $P_{0}^{-1}$ is the information matrix of the a priori estimate and acts as the regularization of the least-squares problem: it is equivalent to a Tikhonov term whose weight is set by the prior covariance, so no separate scalar regularization parameter is needed. For $P_{0}^{-1}\to 0$ the expression reduces to the ordinary weighted least-squares covariance ${\left(A^{T}WA\right)}^{-1}$, which exists only if the measurement geometry makes A of full column rank; the prior keeps weakly observable directions bounded.

The GEO position covariance submatrix for satellite *i* is denoted by $P_{r_{i}}$, such that

(49) $$\delta r_{i}∼N\left(0,P_{r_{i}}\right)$$

The corresponding baseline-error covariance is

(50) $$P_{B}=P_{r_{2}}+P_{r_{1}}-P_{r_{2}r_{1}}-P_{r_{1}r_{2}}$$

where $P_{r_{2}r_{1}}$ and $P_{r_{1}r_{2}}$ are the cross-covariance terms between the two GEO position errors. Equation (50) is the covariance of the difference $\delta r_{2}-\delta r_{1}$, namely Cov(X−Y)=Cov(X)+Cov(Y)−Cov(X,Y)−Cov(Y,X). It remains positive semidefinite, because $P_{B}=\left[I\;\;-I\right]\;P_{\mathrm{joint}}\;{\left[I\;\;-I\right]}^{T}$ with $P_{\mathrm{joint}}$ the joint covariance of the two errors. As for Equation (46), positively correlated errors reduce the baseline covariance and negatively correlated errors increase it. If the two position errors are treated as independent, as in the simulations, the cross-covariance terms vanish and $P_{B}=P_{r_{2}}+P_{r_{1}}$, which is the conservative case.

Published GEO precise orbit-determination studies indicate that sub-meter GEO orbit knowledge is feasible under favorable post-processing and tracking assumptions [28,29,30,31]. Complementary tracking approaches, such as time-difference-of-arrival methods, have also been used to support GEO orbit determination for satellite operators [32]. For RINGS, the key requirement is accurate knowledge of the position of every GEO satellite during the observation arc, because the baseline vector is obtained from these positions. Inter-satellite ranging can refine the along-baseline component but does not replace the orbit determination of the individual satellites.

In the simulations, the orbit-determination covariance of each satellite is represented by an isotropic equivalent, $P_{r_{i}}=\sigma_{\mathrm{ECI}}^{2}I$, i=1,2. Here $\sigma_{\mathrm{ECI}}=\sigma_{x}=\sigma_{y}=\sigma_{z}$ is the per-axis standard deviation of the residual position error of one GEO satellite in Earth-centered inertial (ECI) coordinates, the same for both satellites. For a non-isotropic covariance, the equivalent value is $\sigma_{\mathrm{ECI}}^{2}=\mathrm{tr}\left(P_{r_{i}}\right)/3$. With independent errors, Equation (50) then gives $P_{B}=2\sigma_{\mathrm{ECI}}^{2}I$, that is, a per-axis baseline uncertainty of $\sqrt{2}\;\sigma_{\mathrm{ECI}}$. The examined levels are

(51) $$\sigma_{\mathrm{ECI}}=0.3,\;0.5,\;1,\;2,\;5\;m$$

The 0.3m and 0.5m cases correspond to the sub-meter accuracy reported for precise GEO orbit determination under favorable tracking and post-processing conditions [28,29,30,31], and represent a dedicated RINGS implementation. The 1m and 2m cases represent conservative but still high-quality OD conditions. The 5m case is included as a degraded stress case.

### 2.6. Hardware-Delay Calibration

The internal hardware delays $p_{1}$ and $p_{2}$ in Equations (24) and (25) are a further error source of the timing chain. They are the group delays of the transmit and receive paths between the antenna reference point and the point where the time tag is generated, including cables, filters, amplifiers, frequency converters, analog-to-digital and digital-to-analog conversion, and digital processing. In a software-defined-radio (SDR) implementation they range from a few nanoseconds to hundreds of nanoseconds, depending on the processing complexity, the available computing power, the power constraints on the satellite, and the thermal stability and control of the SDR. Two properties make them manageable. First, they are deterministic and nearly constant: a transponder responds with constant latency, so the delay can be compensated [33]. Second, they can be measured, predicted and calibrated. They are measured by looping a known calibration signal from the transmit chain into the receive chain of the same unit, on the ground before launch and periodically in orbit, so that the differential term $\Delta p_{\mathrm{cal}}$ of Equation (26) is refreshed before each VLBI integration window. In two-way time transfer, SDR receivers are already calibrated together with the conventional modems, and relative delays between receive chains are controlled at the level of tens to about a hundred picoseconds [34]. Measurements on commercial SDRs confirm both sides of this behavior. Once the device has warmed up, the inter-channel offset is stable to a small fraction of a degree, whereas it depends on the carrier frequency, the gain setting and re-tuning and is individual to each device, so that the calibration is repeated after a configuration change [35]. The dependence on temperature and processing load is predictable and can be included in the calibration model.

The importance of this calibration follows from Equation (8). An uncalibrated delay difference of 2ns would enter the two-way estimate as a clock offset and, at the trio baseline, correspond to an angular error of about 8.2nrad, which is larger than the 4nrad performance reference. After calibration, only the residual of the calibration matters. A residual of 30ps corresponds to about 0.12nrad, and the 100ps case simulated in this work is a conservative allowance for calibration residuals, thermal drift between calibrations, and imperfectly modeled propagation terms. Because the hardware-delay residual is a quasi-static bias rather than a stochastic term over the 300s arc, it is represented in the simulations by the constant clock misalignment of Section 3.5. Its practical control is a matter of calibration design, namely the calibration interval, the temperature control of the radio-frequency chain, and the stability of the calibration path itself, and it is not an independent limit on the RINGS concept.

## 3. Simulation and Analysis Methodology

This section describes the numerical simulation used to propagate the clock and GEO position uncertainties through the RINGS measurement chain.

The overall simulation flow is shown in Figure 4. The block diagram illustrates the general RINGS processing chain in terms of the two-dimensional celestial coordinates, Right Ascension (RA) and Declination (Dec), that a multi-baseline implementation of the sensor is ultimately intended to resolve. The numerical analysis in Section 3 and Section 4 evaluates the single-baseline, scalar AOA formulation of Equation (63). An equatorial GEO-GEO baseline of length *B* senses a target at declination δ in RA with a lever arm of order *B* and in Dec with a lever arm of order B|sinδ|. Solar-system targets lie close to the ecliptic, which is inclined by about 23.4° to the equatorial GEO plane, so the scalar AOA mainly represents an RA-type measurement. Dec is also sensed, and for |δ| above about 10° its lever arm exceeds the Earth diameter, which bounds the lever arm of any terrestrial baseline. The reported sensitivities therefore apply directly to RA-type estimation.

All positions, baselines and lines of sight in the simulation are expressed in the ECI frame, taken as the J2000 realization whose axes are parallel to those of the International Celestial Reference Frame (ICRF). The GEO satellites are modeled as circular equatorial orbits in this frame, so that Earth rotation does not enter, and the unit vector to the spacecraft is an ICRF direction. A single baseline constrains this direction to a cone of half-angle θ about the baseline, and RA and Dec follow from two or more non-parallel baselines. The interferometric observable is the phase (equivalently, delay) difference between the two receivers, and the angle is derived from it through the baseline geometry, as in Delta-DOR and terrestrial VLBI. The direction estimate therefore does not rely on the beam pattern or point-spread function of the individual antennas. Antenna directivity enters only through the gain needed for the received signal-to-noise ratio to exceed the receiver threshold. The signal is the narrowband transmission of the cooperating spacecraft, and the absence of receiver-side atmosphere removes the corresponding path delays.

### 3.1. RINGS Measurement Geometry

The two GEO receivers are modeled as circular equatorial GEO satellites with position vectors $r_{1}\left(t\right)$ and $r_{2}\left(t\right)$. The baseline vector B(t) is given by Equation (44), and its length is B(t)=∥B(t)∥. The deep-space spacecraft position is denoted by $r_{\mathrm{sc}}\left(t\right)$, and the line-of-sight unit vector is

(52) $$\hat{s}\left(t\right)=\frac{r_{\mathrm{sc}}\left(t\right)}{∥r_{\mathrm{sc}}\left(t\right)∥}$$

The true angle of arrival relative to the GEO-GEO baseline is

(53) $$\theta \left(t\right)=\cos^{-1}\left[\hat{B}\left(t\right)\cdot \hat{s}\left(t\right)\right]$$

where

(54) $$\hat{B}\left(t\right)=\frac{B(t)}{B(t)}$$

The simulations use two GEO satellites 180° apart, so that B≈84,328km (Equation (10)). Because the sensitivities scale as 1/B (Equation (8)), the results apply to the trio baseline with the factor $B_{\max}/B_{120}\approx 1.15$.

The line of sight is evaluated at each epoch from the spacecraft position $r_{\mathrm{sc}}\left(t\right)$, so the motion of the spacecraft during the 300s arc is part of the model, and the AOA is estimated epoch by epoch rather than assumed constant. The effect of this motion is small compared with the rotation of the baseline. A transverse velocity $v_{⊥}$ relative to the Earth at distance *R* changes the direction to the spacecraft by about $v_{⊥}T/R$, which is $6\times 10^{-5}\;\mathrm{rad}$ over T=300s for $v_{⊥}=30\;\mathrm{km}\;s^{-1}$ at 1au, whereas the GEO-GEO baseline rotates by $\omega_{\mathrm{GEO}}T\approx 2.2\times 10^{-2}\;\mathrm{rad}$. Both changes are deterministic. In the navigation solution, the spacecraft motion is reconstructed by the dynamical model of the orbit-determination estimator described in Section 1.

### 3.2. Signal and Phase Observable Model

The received complex baseband field at GEO receiver *m* is modeled as

(55) $$E_{m}\left(t\right)=A_{m}\left(t\right)e^{j\phi_{m}\left(t\right)}+n_{m}\left(t\right),\;m=1,2$$

where $A_{m}\left(t\right)$ is amplitude, $\phi_{m}\left(t\right)$ is carrier phase, and $n_{m}\left(t\right)$ is receiver noise. The measured phase difference is

(56) $$\Delta \phi \left(t\right)=\phi_{2}\left(t\right)-\phi_{1}\left(t\right)$$

In the plane-wave approximation, for a narrowband signal,

(57) $$\Delta \phi \left(t\right)=\frac{2\pi }{\lambda }B\left(t\right)\cdot \hat{s}\left(t\right)$$

The estimator adopts the standard plane-wave interferometric model of Equation (57), in which the differential phase depends only on the baseline projection along the line of sight. The simulated phases, however, are computed from the exact ranges to the target, so the simulation retains the wavefront curvature. This is the same simplified closed-form solution of the wave equation conventionally used in ground-based VLBI and Delta-DOR processing. Even terrestrial baselines, much shorter than the GEO-GEO baseline considered here, do not strictly satisfy classical far-field criteria either, and the approximation is retained there for the same reason. For a source at distance *R*, the wavefront curvature adds to the path difference a term of order $B^{2}\sin^{2}\theta /\left(2R\right)$, about 18km for B=73,000km at 1au. This term is large but deterministic. It is computed from the a priori spacecraft range, as in finite-distance VLBI delay models [36], and an error δR in that range leaves a residual of order $(B^{2}/2R^{2})\;\delta R$, about 0.1mm, or $10^{-12}\;\mathrm{rad}$ in angle, at 1au for δR=1km. The term is larger for closer targets and is modeled in the same way. For the simulated antipodal pair the leading term cancels by symmetry, and the next term, $\left(\hat{s}\cdot r_{1}\right)\left(r_{\mathrm{GEO}}^{2}-{\left(\hat{s}\cdot r_{1}\right)}^{2}\right)/R^{2}\approx 2.7\;m$ at $R=10^{8}\;\mathrm{km}$, appears as the constant offset of about 44nrad discussed in Section 4.2. It is therefore removed in the operational processing chain and does not contribute to the stochastic precision floor analyzed here.

Using

(58) $$B\left(t\right)\cdot \hat{s}\left(t\right)=B\left(t\right)\cos\theta \left(t\right)$$

the phase observable becomes

(59) $$\Delta \phi \left(t\right)=\frac{2\pi }{\lambda }B\left(t\right)\cos\theta \left(t\right)$$

The corresponding angle estimate is

(60) $$\hat{\theta }\left(t\right)=\cos^{-1}\left[\frac{\lambda }{2\pi \hat{B}\left(t\right)}\hat{\Delta \phi }\left(t\right)\right]$$

### 3.3. Dual-Frequency Angle-of-Arrival Reconstruction

The simulation uses a dual-frequency wide-lane observable, whose purpose is to widen the unambiguous delay interval as introduced in Section 1 and Section 2.1. For two carrier frequencies $f_{1}$ and $f_{2}$, with

(61) $$\Delta f_{c}=f_{2}-f_{1}$$

the differential phase observable is

(62) $$\Delta \Delta \phi \left(t\right)=\frac{2\pi B(t)}{c}\Delta f_{c}\cos\theta \left(t\right)$$

The angle estimate is therefore

(63) $$\hat{\theta }\left(t\right)=\cos^{-1}\left[\frac{c}{2\pi \hat{B}\left(t\right)\Delta f_{c}}\hat{\Delta \Delta \phi }\left(t\right)\right]$$

Equations (60) and (63) apply the same inverse-cosine mapping to different observables. In Equation (60) it is the inter-receiver phase difference at one carrier, with scale λ=c/f, so one cycle of ambiguity corresponds to an angle of order λ/B. In Equation (63) it is the double difference $\Delta \Delta \phi =\Delta \phi \left(f_{2}\right)-\Delta \phi \left(f_{1}\right)$, with the much longer wide-lane scale $c/\Delta f_{c}$, which widens the ambiguity interval. Equation (63) is the estimator used in the Monte Carlo simulations.

### 3.4. Monte Carlo Statistical Metrics

The variance-based metrics defined below characterize the statistical precision of the RINGS AOA estimator, that is, the dispersion of repeated estimates under a given clock and GEO position uncertainty. They do not characterize the absolute accuracy after calibration, which also depends on deterministic effects such as residual hardware-delay miscalibration or antenna phase-center offsets. These effects are assumed to be removed separately through dedicated calibration.

For $N_{\mathrm{MC}}$ Monte Carlo realizations, the angle estimate in realization *k* is denoted by ${\hat{\theta }}_{k}\left(t\right)$. The ensemble mean is

(64) $$\bar{\theta }\left(t\right)=\frac{1}{N_{\mathrm{MC}}}\sum_{k=1}^{N_{\mathrm{MC}}}{\hat{\theta }}_{k}\left(t\right)$$

The angular standard deviation is

(65) $$\sigma_{\theta }\left(t\right)=\sqrt{\frac{1}{N_{\mathrm{MC}}-1}\sum_{k=1}^{N_{\mathrm{MC}}}{\left[{\hat{\theta }}_{k}\left(t\right)-\bar{\theta }\left(t\right)\right]}^{2}}$$

For each Monte Carlo run, the RMS residual over the full observation arc is

(66) $${\mathrm{RMS}}_{k}=\sqrt{\frac{1}{N_{t}}\sum_{i=1}^{N_{t}}{\left[{\hat{\theta }}_{k}\left(t_{i}\right)-\theta_{\mathrm{true}}\left(t_{i}\right)\right]}^{2}}$$

This metric is particularly useful when the dominant uncertainty is quasi-static over the short observation arc.

### 3.5. Clock-Bias Perturbation Model

The residual relative clock offset in Monte Carlo realization *k* is modeled as

(67) $$\delta t_{k}∼N\left(0,\sigma_{t}^{2}\right)$$

For the dual-frequency observable, the corresponding phase perturbation is

(68) $$\Delta \Delta \phi_{\mathrm{clk},k}=2\pi \Delta f_{c}\delta t_{k}$$

The clock offset is treated as constant over each 300s observation arc. This represents a stable residual synchronization bias over a short observation arc. The clock cases examined are

(69) $$\sigma_{t}=7,\;10,\;50,\;100,\;300\;\mathrm{ps}$$

An additional 1000ps case is included in the combined position-clock sweep to test degraded timing sensitivity.

### 3.6. GEO Position-Perturbation Model

The nominal GEO position vector of satellite *m* in the ECI frame is

(70) $$r_{m}^{\mathrm{ECI}}\left(t\right)=\left[\begin{array}{c} x_{m}\left(t\right)\\ y_{m}\left(t\right)\\ z_{m}\left(t\right) \end{array}\right]$$

A random Cartesian position perturbation is applied as

(71) $${\tilde{r}}_{m}^{\mathrm{ECI}}\left(t\right)=r_{m}^{\mathrm{ECI}}\left(t\right)+\eta_{r,m}$$

where

(72) $$\eta_{r,m}=\left[\begin{array}{c} \eta_{x,m}\\ \eta_{y,m}\\ \eta_{z,m} \end{array}\right]∼N\left(0,\Sigma_{r}\right)$$

and

(73) $$\Sigma_{r}=\mathrm{diag}\left(\sigma_{x}^{2},\sigma_{y}^{2},\sigma_{z}^{2}\right)$$

In the absence of mission-specific GEO orbit-determination covariance data, the main three-dimensional cases model the GEO position uncertainty as isotropic Cartesian uncertainty in the ECI frame:

(74) $$\sigma_{x}=\sigma_{y}=\sigma_{z}=\sigma_{\mathrm{ECI}}$$

For low-declination observation geometries, a reduced two-dimensional approximation is also considered:

(75) $${\tilde{r}}_{m,2D}^{\mathrm{ECI}}\left(t\right)=\left[\begin{array}{c} x_{m}\left(t\right)\\ y_{m}\left(t\right) \end{array}\right]+\left[\begin{array}{c} \eta_{x,m}\\ \eta_{y,m} \end{array}\right]$$

with

(76) $$\left[\begin{array}{c} \eta_{x,m}\\ \eta_{y,m} \end{array}\right]∼N\left(0,\mathrm{diag}\left(\sigma_{x}^{2},\sigma_{y}^{2}\right)\right)$$

### 3.7. Combined Position-Clock Simulation Model

The combined position-clock sweep evaluates the joint sensitivity of the RINGS estimator to GEO position uncertainty and relative clock misalignment. In the combined plot, the horizontal axis is the total three-dimensional position RMS, ${\mathrm{RMS}}_{3D}=\sqrt{\sigma_{x}^{2}+\sigma_{y}^{2}+\sigma_{z}^{2}}$, rather than the per-axis standard deviation used in Section 2.5 and in the position-only sweep of Section 4.2. The corresponding per-axis Cartesian standard deviation is

(77) $$\sigma_{x}=\sigma_{y}=\sigma_{z}=\frac{{\mathrm{RMS}}_{3D}}{\sqrt{3}}$$

The combined simulation redraws the position errors at every epoch and uses common normalized random samples across all tested position and clock levels. This reduces Monte Carlo draw-to-draw variation and makes the ordering of the curves reflect the physical sensitivity to the uncertainty level. The parameters of the Monte Carlo simulations, their ranges, and the reasons for the chosen values are summarized in Table 4.

## 4. Simulation Results and Analysis

This section presents the Monte Carlo results for clock errors, GEO position errors, and their combination.

### 4.1. Clock-Error Simulation Results

The clock-error simulation evaluates the sensitivity of the RINGS AOA estimator to residual inter-satellite timing uncertainty while setting GEO position uncertainty to zero. Each Monte Carlo realization contains one stable relative timing offset over the 300s observation arc.

Rather than plotting $\sigma_{\theta }\left(t\right)$ directly for a small number of clock cases, the clock sensitivity is summarized by averaging the angular standard deviation over the full 300s integration window. The clock uncertainty is swept from 5ps to 100ps in 5ps increments. Figure 5 shows the resulting mean angular scatter as a function of clock misalignment. The upper panel shows the averaged Monte Carlo result and a linear fit, while the lower panel shows the residual from the linear fit.

Figure 5 shows that the clock-only sensitivity is nearly linear over the examined range. The fitted slope is approximately 0.00513nrad/ps, with $R^{2}\approx 0.999$. This behavior is expected in the small-error regime because a residual timing error produces a phase perturbation proportional to δt, and the angle estimator maps this perturbation approximately linearly as long as the solution remains within the local phase branch. The fitted slope is somewhat steeper than the characteristic sizing estimate of Equation (8), which gives approximately 0.00356nrad/ps at the simulated baseline. This is expected. As noted in Section 2.1, the closed-form relation captures the *B*-dependence of the clock sensitivity but not the order-unity geometric factor 1/sinθ(t) set by the actual, time-varying baseline-to-line-of-sight geometry. With θ≈45° over the simulated arc this factor is about 1.41, which gives 0.0050nrad/ps, in agreement with the simulation.

The upper panel is a straight line because the clock-to-angle mapping is linear in the small-error regime, and the fitted slope is the sensitivity coefficient of interest. A logarithmic scale is not used because a proportional relation appears as a straight line of unit slope in logarithmic axes as well, so the departures from linearity are better shown by the residual panel. The residuals in the lower panel are small, remaining at the level of a few $10^{-3}\;\mathrm{nrad}$ to $10^{-2}\;\mathrm{nrad}$. These residuals are consistent with finite Monte Carlo sampling, time averaging over a slowly changing geometry, and weak nonlinearity of the inverse $\cos^{-1}$ angle reconstruction. They should not be interpreted as a phase-ambiguity signature.

Table 5 summarizes selected representative clock-misalignment cases, namely the cases of Equation (69). These values are consistent with the fine sweep shown in Figure 5 and provide discrete reference points for comparison with the clock-stability analysis.

Even 300ps gives only about 1.5nrad, so after calibration of deterministic bias and drift the residual stochastic clock term is not expected to dominate the RINGS error budget.

### 4.2. GEO Position-Uncertainty Impact Simulation Results

The GEO position-uncertainty simulations evaluate the sensitivity of the RINGS AOA estimator to residual GEO position errors, applied independently to the two satellites with the isotropic model of Section 3.6.

Two representative reconstruction cases were first examined. The first is a high-accuracy case with 0.5m per-axis GEO position uncertainty and 10ps clock misalignment. The second is a conservative degraded case with 5m per-axis GEO position uncertainty and 100ps clock misalignment. These two cases bracket the examined range of GEO orbit-determination accuracy and are used to illustrate the time-domain behavior of the reconstructed RINGS AOA. All five position levels of Equation (51) were simulated.

Figure 6 shows that the reconstructed RINGS AOA follows the true AOA closely over the 300s observation arc. The residuals are nearly constant over the arc, as expected for quasi-static position and clock errors, and they are at the level of tens of nanoradians for the 0.5m case, not at the nanoradian level. The mean of the residual is a deterministic offset of about 44nrad, the wavefront-curvature term of the plane-wave estimator (footnote to Equation (57)). In practice such deterministic terms, like calibration residuals, are computed or calibrated and removed. What cannot be removed is the stochastic scatter of the estimates, which is set by the position and clock uncertainties and by the stochastic nature of the observable. For the 0.5m case the ±1σ scatter is about 8nrad. The aim of these simulations is to verify the trends of this scatter with position and clock uncertainty, and the sensitivity results below are therefore based on the ensemble standard deviation $\sigma_{\theta }\left(t\right)$ of Equation (65), which does not depend on a constant offset.

Figure 7 shows the degraded case. The residual spread increases, but the estimator remains stable and follows the true AOA without divergence.

The per-run RMS residuals for the two representative reconstruction cases are shown in Figure 8. This metric captures the accumulated effect of quasi-static position and clock residuals over the full observation arc. Because it is an RMS about the truth, it contains both the quasi-static offset and the stochastic scatter, and the scatter is the component of interest.

The conservative case produces a wider residual distribution and larger outliers, as expected, but no estimator divergence over the examined range.

To quantify the dependence on GEO position uncertainty more directly, a position-only sensitivity sweep was also performed by setting the clock perturbation to zero. The position sensitivity is summarized by averaging $\sigma_{\theta }\left(t\right)$ over the full 300s integration window. The GEO coordinate uncertainty is swept from 0.5m to 5m per axis in 0.5m increments. Figure 9 shows the resulting averaged angular scatter as a function of GEO position uncertainty. The upper panel shows the averaged Monte Carlo result and linear fit, while the lower panel shows the residual from the linear fit.

Figure 9 shows a nearly linear dependence of angular scatter on GEO coordinate uncertainty. The fitted slope is approximately 16.94nrad/m, with $R^{2}\approx 0.9996$, when the horizontal axis is interpreted as the per-axis ECI coordinate uncertainty $\sigma_{x}=\sigma_{y}=\sigma_{z}$. It agrees with the first-order estimate $\sqrt{2}/B\approx 16.8\;\mathrm{nrad}/m$, since the baseline error of the two independent satellites has a per-axis standard deviation of $\sqrt{2}\;\sigma_{\mathrm{ECI}}$. At the trio baseline the slope is about 19.6nrad/m. This slope, discussed further in Section 5.1, identifies GEO position knowledge as the dominant performance driver among the error sources considered in this study.

The residuals from the linear fit remain below approximately 1nrad across the examined range. These residuals are not indicative of phase ambiguity. They are consistent with finite Monte Carlo sampling, the nonlinear transformation from baseline projection to angle through the inverse $\cos^{-1}$ estimator, and the fact that the sensitivity of the baseline projection varies slightly with geometry over the 300s arc. The dominant first-order behavior remains linear, as expected from the perturbation of the GEO-GEO baseline vector.

Sub-meter GEO position uncertainty is compatible with nanoradian-level performance, while several-meter uncertainty leads to a larger but still bounded AOA scatter.

### 4.3. Combined Position-Clock Sensitivity

The combined sensitivity analysis evaluates the joint effect of GEO position uncertainty and residual relative clock misalignment. Unlike the position-only sweep in Section 4.2, position uncertainty here is reported as the three-dimensional RMS, ${\mathrm{RMS}}_{3D}=\sqrt{\sigma_{x}^{2}+\sigma_{y}^{2}+\sigma_{z}^{2}}$, following Equation (77). Table 6 summarizes representative values from the combined sweep.

The combined results show that GEO position uncertainty dominates once the position RMS reaches the meter level. Residual clock misalignment remains visible primarily in the low-position-uncertainty regime. For example, at 0.5m position RMS, increasing the clock misalignment from 10ps to 1000ps increases the mean angular scatter from 4.85nrad to 6.94nrad. At 5m position RMS, the same clock degradation changes the mean scatter only from 48.45nrad to 48.69nrad.

Figure 10 highlights the low-position-uncertainty regime, where residual clock misalignment has a visible contribution. As position uncertainty increases, the clock curves converge, indicating that further clock improvement provides diminishing benefit unless GEO position uncertainty is also reduced. This confirms that, once clock stability reaches the picosecond to tens-of-picoseconds regime, GEO position knowledge becomes the dominant performance driver among the error sources considered in this study.

## 5. Discussion

The two central questions of this work are whether the GEO positions, and hence the GEO-GEO baseline vector, and the inter-satellite clock alignment can be known well enough to support nanoradian-class angle-of-arrival estimation. The results indicate that both requirements are demanding, but not prohibitive.

### 5.1. Removal of the Main Feasibility Barriers

The main feasibility concern for RINGS is whether moving GEO satellites can serve as sufficiently well-known interferometric stations. Unlike terrestrial VLBI, where ground-station coordinates are known with very high accuracy, the critical quantity is the knowledge of the position of each GEO satellite during the observation arc, since both positions together define the baseline vector that enters the interferometric phase. A precisely known baseline length is not sufficient: if the satellite positions are wrong, the baseline direction is wrong and the measured phase is mapped into a wrong angle.

The position-only sweep in Figure 9 translates this requirement into a direct sensitivity of approximately 16.94nrad/m per axis. Sub-meter position knowledge is therefore compatible with terrestrial-VLBI-order nanoradian performance, while several-meter uncertainty gives tens of nanoradians. GEO position knowledge is thus the dominant practical barrier, but not a prohibitive one. The requirement is not centimeter-level real-time positioning, but sub-meter to meter-level knowledge of each satellite over a short arc, which can be supported by high-end GEO orbit determination, post-processed orbit and baseline estimation, and dedicated calibration, with inter-satellite ranging as an additional constraint on the along-baseline component.

The second obstacle, inter-satellite clock alignment, has two roles. The first is short-term stochastic stability over the integration window. With the PHM-class clocks of Section 2.2 it is at the 10ps level, which contributes only about 0.05nrad (Figure 5). The second is the removal of deterministic or slowly varying bias, such as clock offset, aging, calibration residuals and drift, which would otherwise appear directly as a phase bias. The clock is therefore not the dominant stochastic error source after calibration, but it remains an essential enabling subsystem that requires high-stability atomic clocks, regular calibration, and two-way inter-satellite time transfer.

The combined sweep confirms this ranking. Once the position uncertainty reaches the meter level it dominates the total scatter, and further clock improvement gives diminishing benefit unless the position uncertainty is also reduced.

### 5.2. Implications for Practical RINGS Implementation

The practical design of RINGS should focus on accurate position knowledge of the GEO satellites and on a stable phase reference over the few-minute observation window. The 300s arc used here is short enough for clock drift and GEO dynamics to be treated as slowly varying residuals, and long enough for coherent phase collection at both GEO receivers.

A practical implementation therefore needs four dedicated elements. First, precise orbit determination of each GEO satellite, with suitable navigation aids, accurate dynamic modeling and tracking, and preferably inter-satellite ranging as an additional constraint on the baseline; the slope of Figure 9 shows why this requirement is central. Second, a payload RF antenna directed toward the deep-space spacecraft with enough gain for phase extraction, together with reliable Doppler correction. Antenna size and receive-chain sensitivity are direct design drivers, although the present sweeps isolate only the position and clock mechanisms. Third, a timing subsystem with a high-stability atomic clock (Section 2.2), a calibration concept for long-term bias and aging, and an inter-satellite link for two-way time and frequency transfer that also contributes to baseline ranging. Fourth, a ground segment for command, telemetry, and the transfer of observation, calibration, timing and orbit-determination data to a processing center, where phase, clock products, GEO positions and ambiguities are estimated jointly. Deep-space navigation dynamics allow this processing over minutes to hours.

### 5.3. Phase Ambiguity Considerations

Phase ambiguity is a common challenge in long-baseline interferometry and is also expected in RINGS. In the present analysis, it is assumed to be resolved by a parallel processing effort based on the multi-frequency principle of Section 1: the wide-lane combination of two carriers provides a coarse, unambiguous delay, and additional carrier frequencies and receiving stations, such as the third satellite of the GEO trio, make the remaining candidate solutions distinguishable. The clock-only and position-only results are therefore interpreted within the local small-error regime, where the estimator remains on the correct phase branch. A complete treatment of phase-ambiguity resolution is left to later stages of the RINGS development.

### 5.4. The Two-Station RINGS Sensor and the Three-GEO Architecture

RINGS is a two-station sensor: each AOA measurement comes from one GEO-GEO baseline, and the trio does not change this physics. The trio used throughout the paper is preferred over a single pair for system reasons: more satellite pairs have the inter-satellite line of sight needed for the two-way link of Section 2.3, the loss of one satellite or link still leaves a usable baseline, and at least one baseline usually keeps an unobstructed view of the target. The price is small. The trio baseline $B_{120}\approx 73,000\;\mathrm{km}$ (Equation (9)) is only 13.4% shorter than $B_{\max}\approx 84,300\;\mathrm{km}$ (Equation (10)), which raises the simulated angular errors of Section 4 by about 15%.

The trio is the minimal configuration that demonstrates the principle, placed in the geostationary belt where platforms are widely deployed and station keeping is routine, and it can grow into a larger network. Additional GEO satellites add redundancy, coverage, and baselines of different lengths that improve phase-ambiguity resolution. Because the GEO belt lies in the equatorial plane, a GEO-only network senses the declination of targets near the celestial equator less strongly than their RA (Section 3). Combining the GEO sensors with sensors in low and medium Earth orbits, in halo orbits, or mounted on the Moon would add baseline components out of the equatorial plane and, for halo and lunar sensors, much longer baselines. Quantifying these benefits is left to future work.

## 6. Conclusions

This paper extended the RINGS space-based VLBI concept introduced in [4] with a time-position error analysis of its fundamental sensor, a two-station GEO-based deep-space angular sensor. The main quantitative results, obtained for a GEO pair with B≈84,300km and about 15% larger at the trio baseline of 73,000km, are the following.

- Residual clock misalignment maps linearly into angular scatter with a sensitivity of 0.00513nrad/ps: a 10ps residual gives about 0.05nrad and a 100ps residual about 0.5nrad. Passive hydrogen maser clocks keep the short-term relative timing at about 10ps over the 300s arc, whereas GNSS-based synchronization alone would contribute about 5.8nrad.
- GEO position uncertainty maps linearly into angular scatter with a sensitivity of 16.94nrad/m per axis, so a per-axis uncertainty of 0.3m gives about 5nrad and 5m gives about 85nrad. Position knowledge of each satellite is therefore the dominant requirement.
- In the two-way time transfer, the Sagnac-type asymmetry biases the uncorrected clock-offset estimate by about 1.25μs and must be computed from the GEO states, which requires knowledge of the inertial velocity of each satellite at the $\mathrm{mm}\;s^{-1}$ level. The Shapiro delay (about 78ps) and the hardware delays (nanoseconds to hundreds of nanoseconds) are deterministic and are removed by modeling and calibration.

As noted in Section 3.4, these figures characterize the statistical precision floor of the two-station sensor rather than its post-calibration accuracy. With sub-meter GEO position knowledge and PHM-class, two-way-synchronized timing, this precision floor reaches the few-nanoradian level of terrestrial VLBI. These capability levels are not merely assumed: PHM-class clocks are already flight-qualified for satellite navigation, and the required GEO position-knowledge levels are consistent with published precise-orbit-determination performance.

Overall, these results increase confidence in the feasibility of RINGS as a complementary space-based VLBI navigation sensor. The central question of this study, whether GEO-based VLBI can plausibly reach nanoradian-class angular precision, is answered in the affirmative for the two dominant error sources examined here.

Future work will focus on: (i) system engineering of a dedicated RINGS GEO platform, including precise orbit determination, a deep-space-directed RF antenna, an onboard atomic reference clock, and an inter-satellite link supporting two-way time transfer and baseline ranging; (ii) robust phase-ambiguity resolution for long-baseline RINGS interferometry; (iii) extension of the error model to realistic measurement noise, link-budget effects, non-isotropic and time-correlated orbit-determination covariance, and Doppler dynamics; and (iv) navigation-filter-level trajectory estimation using the RINGS angle-of-arrival observable.

## Figures and Tables

**Figure 1:**
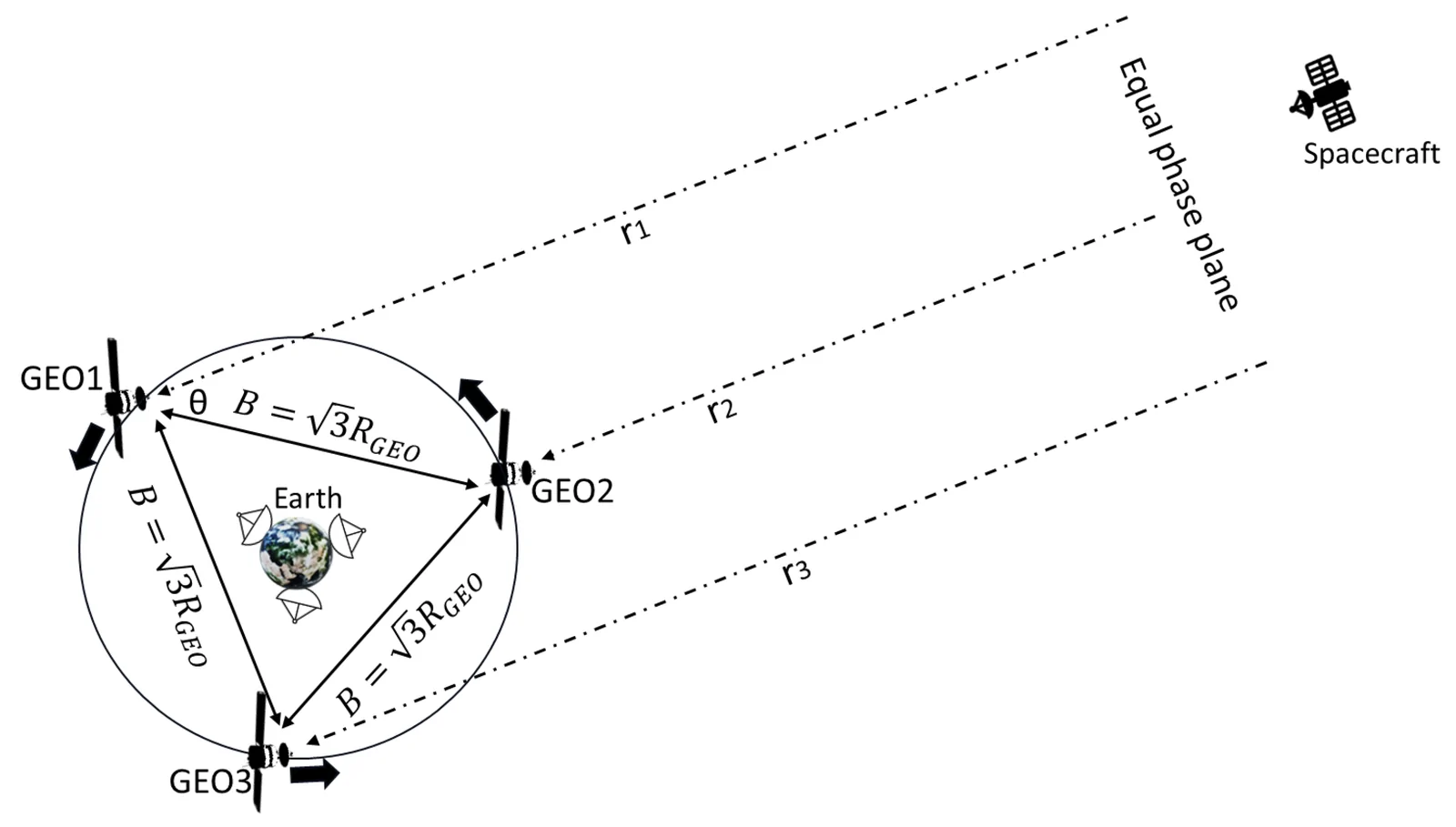
RINGS configuration with three GEO satellites separated by approximately 120°.

**Figure 2:**
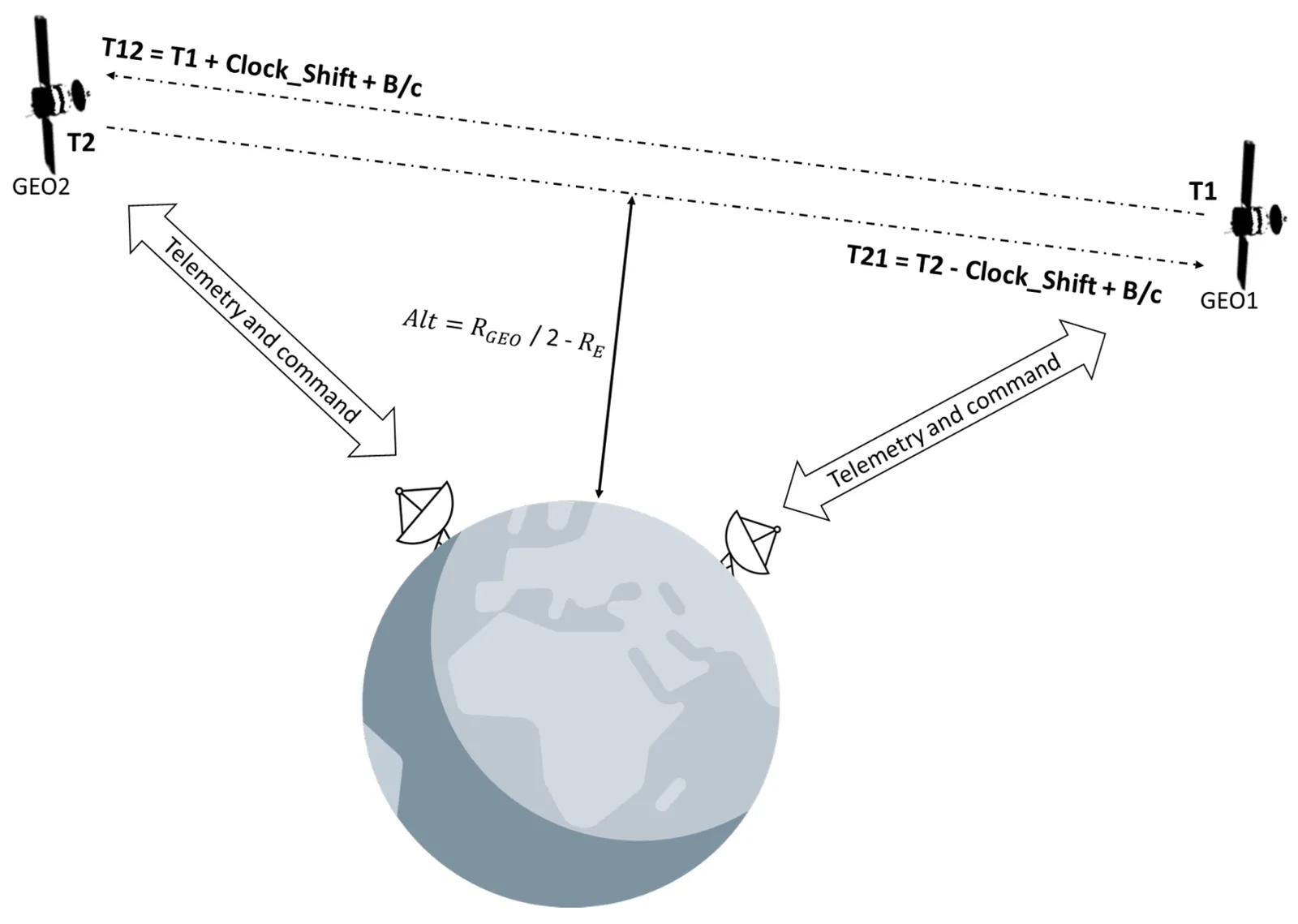
Two-way time and frequency transfer implemented in the RINGS system using a bidirectional inter-satellite link.

**Figure 3:**
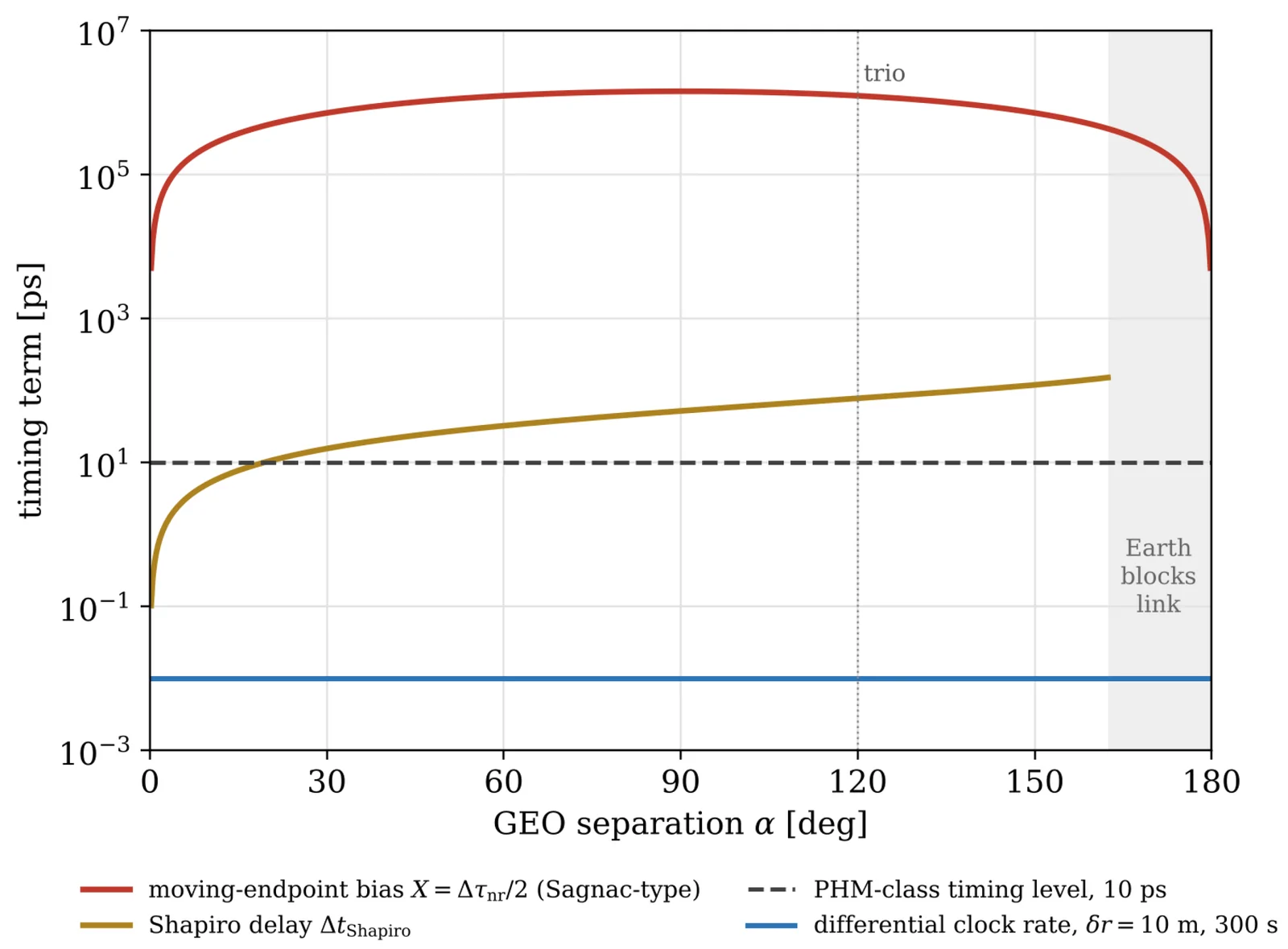
Size of the three timing terms of Section 2.4 versus the GEO-GEO separation α: the moving-endpoint bias *X* of the uncorrected two-way clock-offset estimate (Equation (42)), the Shapiro delay (Equation (40)), shown up to the separation at which the Earth blocks the link, and the differential clock-rate effect over 300s for a 10m radial mismatch (Equation (39)), compared with the 10ps PHM-class timing level (Equation (23)).

**Figure 4:**
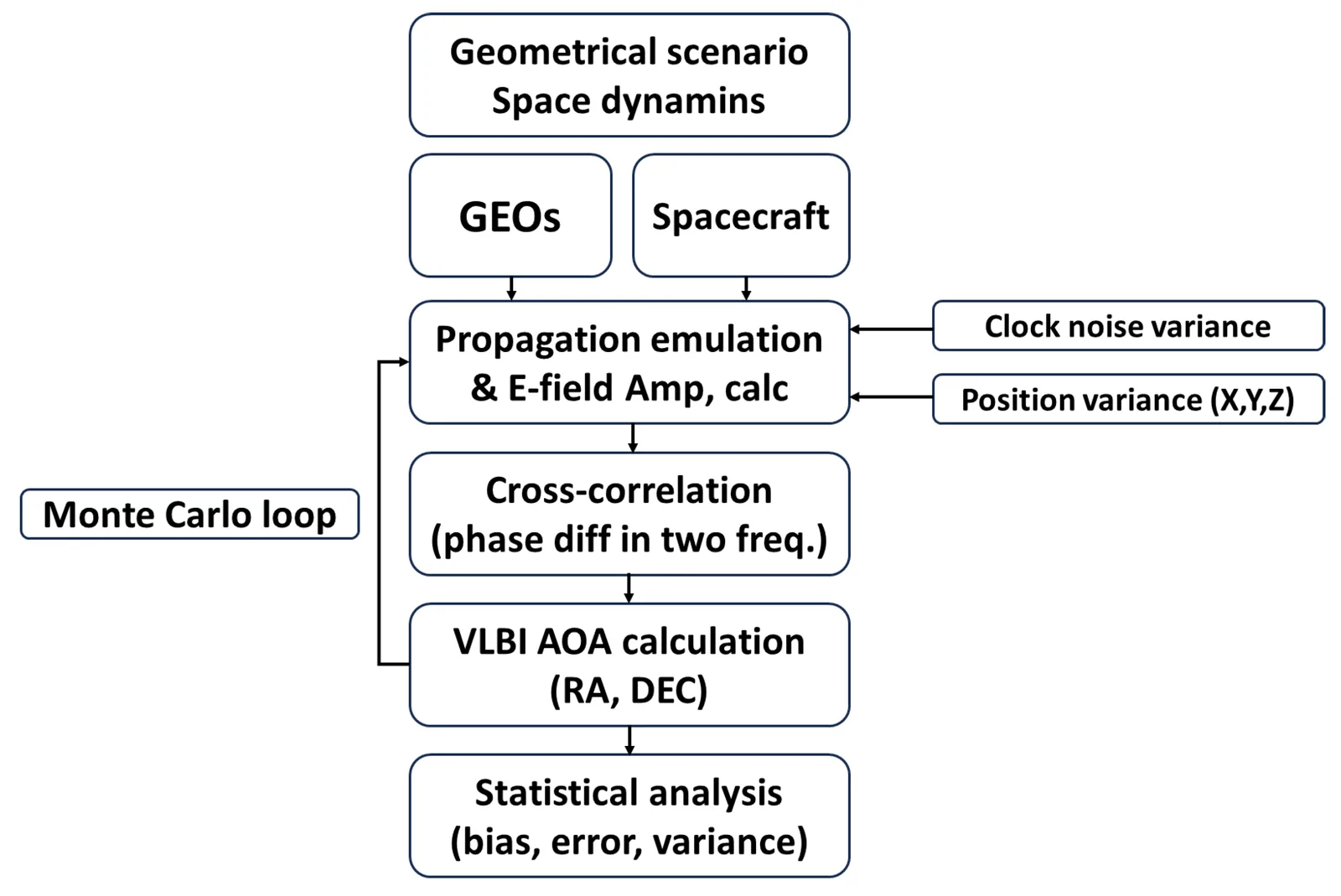
RINGS simulation-based performance-analysis flow.

**Figure 5:**
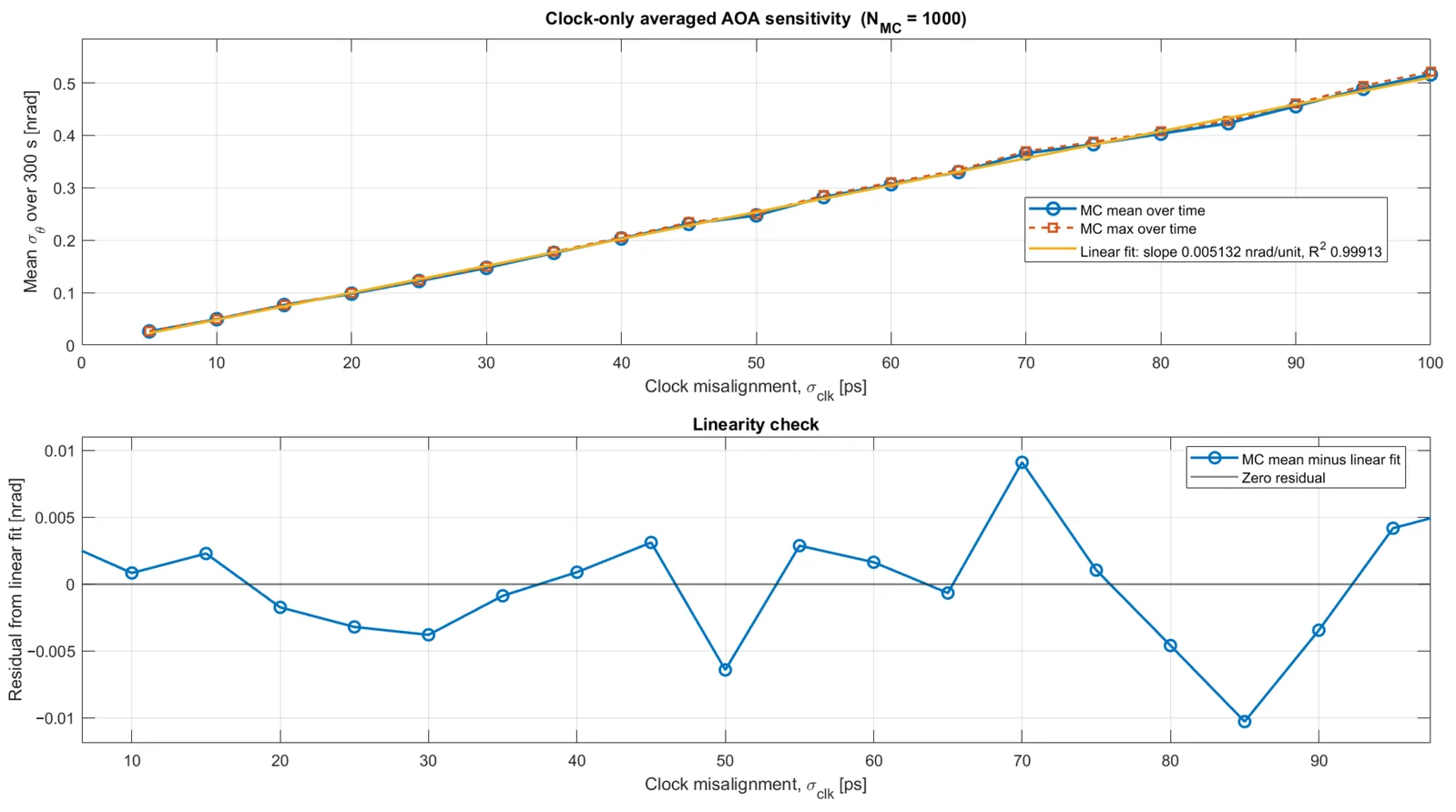
Clock-only averaged AOA sensitivity. The upper panel shows the mean AOA standard deviation over the 300s arc as a function of residual relative clock misalignment. The straight line is the least-squares linear fit, and its slope is the clock sensitivity coefficient; the Monte Carlo points and the fit nearly coincide in the upper panel, which is the expected result. The differences are shown in the lower panel, which plots the residual from the linear fit on an expanded scale.

**Figure 6:**
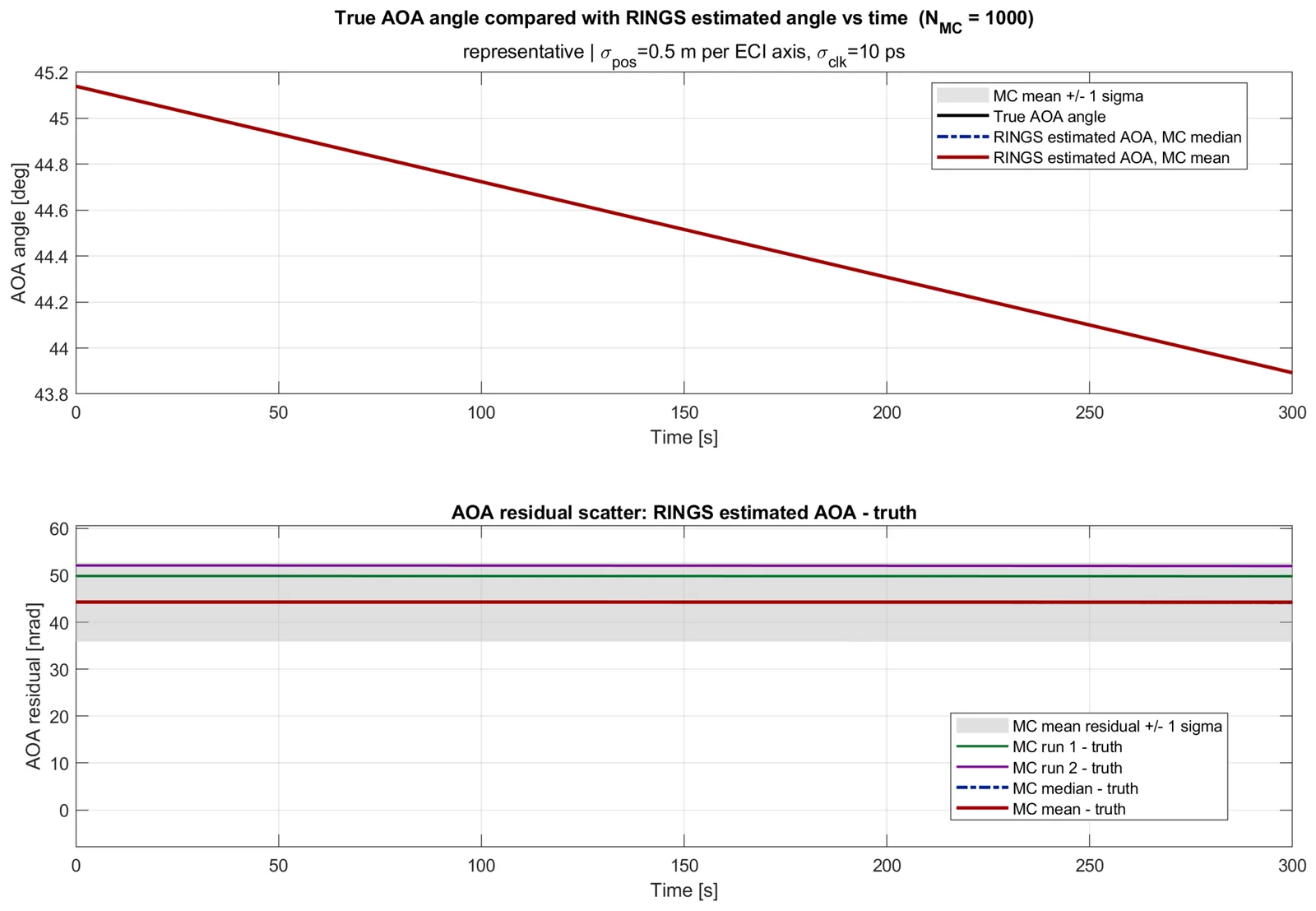
RINGS AOA reconstruction for the high-accuracy case of 0.5m per-axis GEO position uncertainty and 10ps clock misalignment. (**Upper panel**): true AOA and RINGS estimated AOA (Monte Carlo mean and median) versus time. The curves coincide because the AOA changes by about 1.3° (roughly 22mrad) over the arc, more than five orders of magnitude more than the estimation error. (**Lower panel**): residual (estimated minus true) in nrad. “MC run 1” and “MC run 2” are the first and the second of the $N_{\mathrm{MC}}=1000$ Monte Carlo realizations, shown as two typical single runs. “MC mean” and “MC median” are the ensemble mean and median of the residual over all 1000 runs; the median curve lies almost exactly under the mean curve. The gray band is the ensemble mean ±1σ.

**Figure 7:**
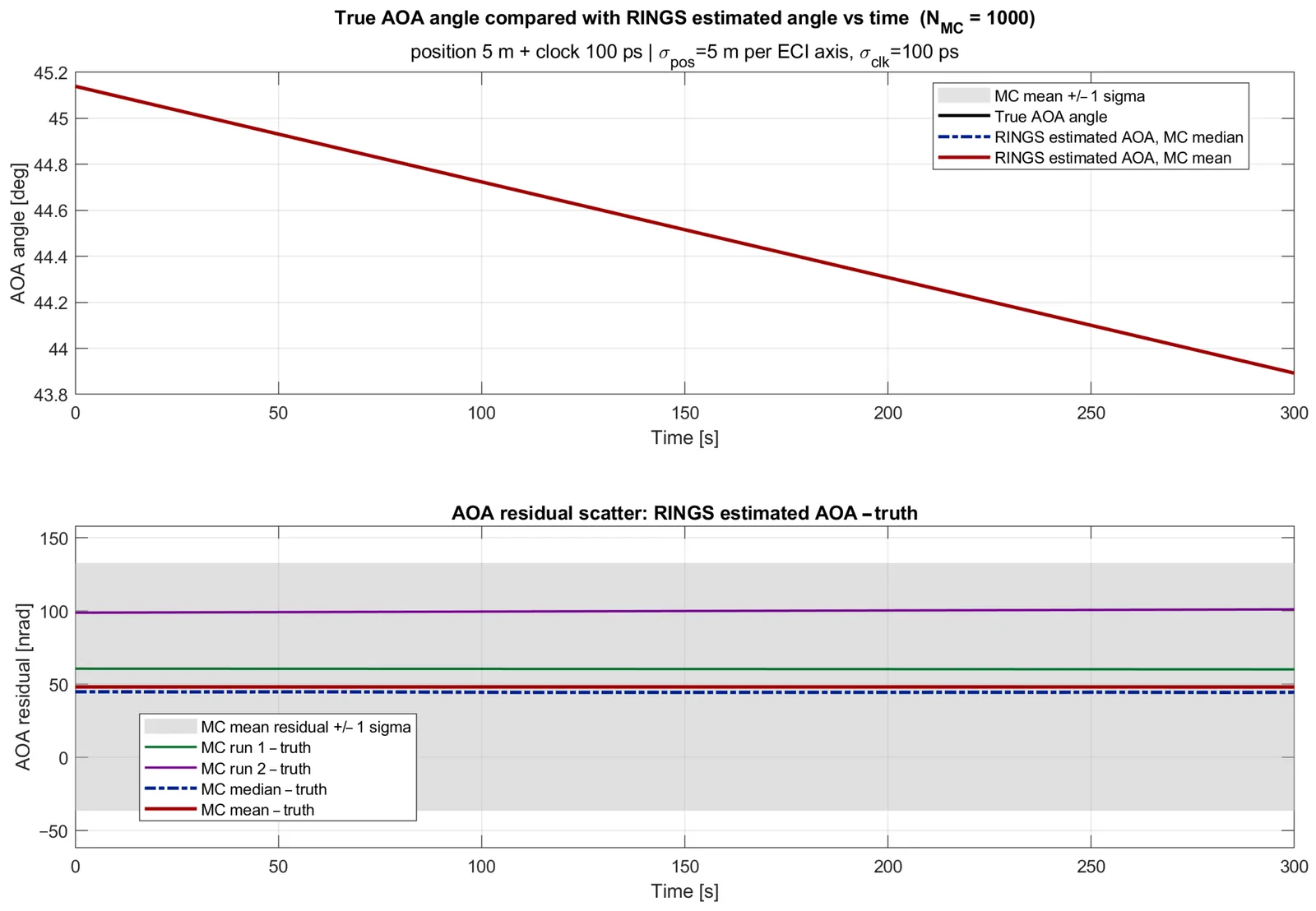
RINGS AOA reconstruction for the conservative case of 5m per-axis GEO position uncertainty and 100ps clock misalignment. Panels and curves as in Figure 6: MC run 1 and MC run 2 are the first two of the 1000 realizations, and the MC median curve lies almost under the MC mean curve.

**Figure 8:**
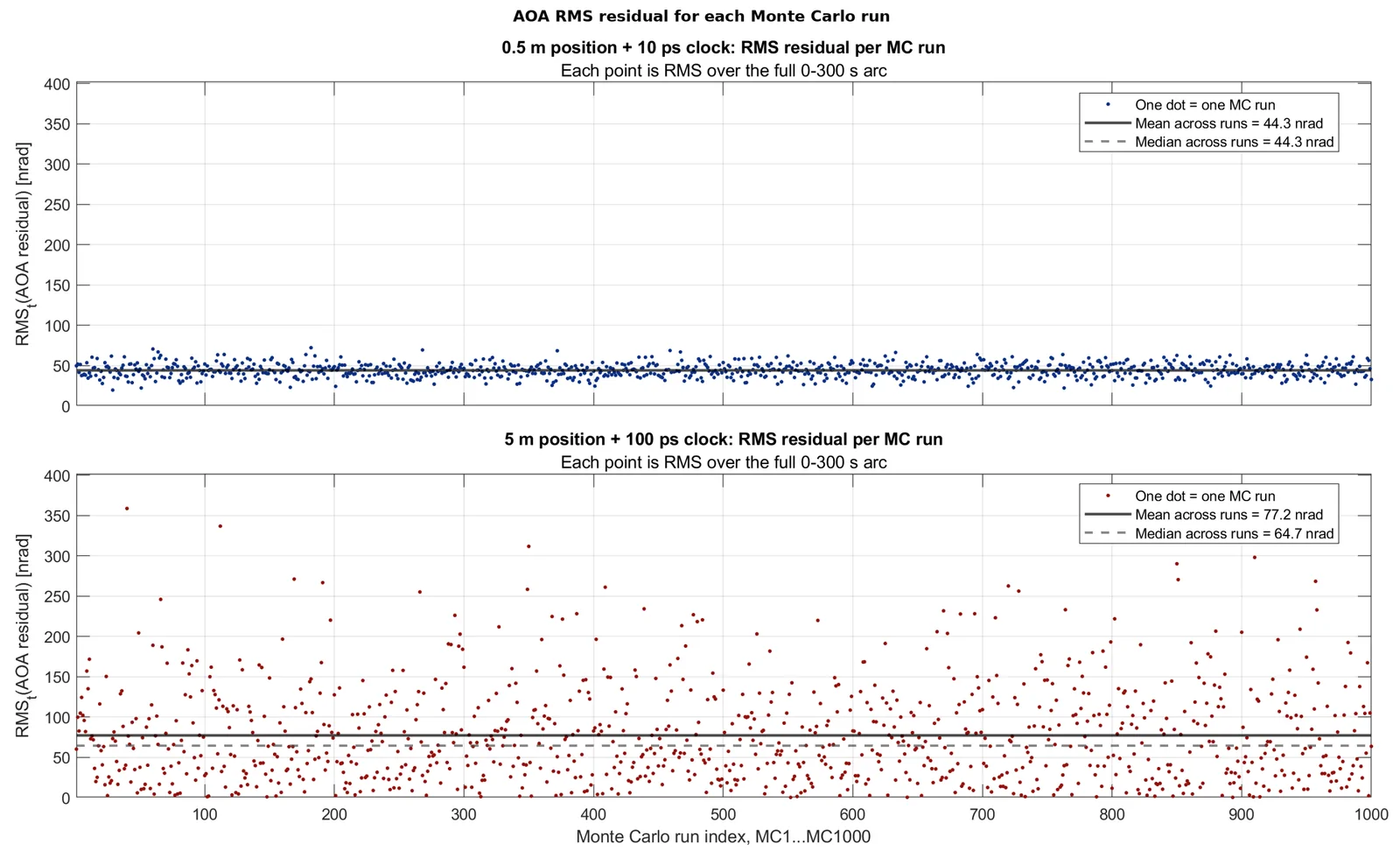
RMS AOA residual for each of the 1000 Monte Carlo runs. (**Upper panel**): 0.5m per-axis position uncertainty and 10ps clock misalignment. (**Lower panel**): 5m per-axis position uncertainty and 100ps clock misalignment. Each point is the RMS residual of one run over the full 300s arc; the solid and dashed lines are the mean and median over the runs.

**Figure 9:**
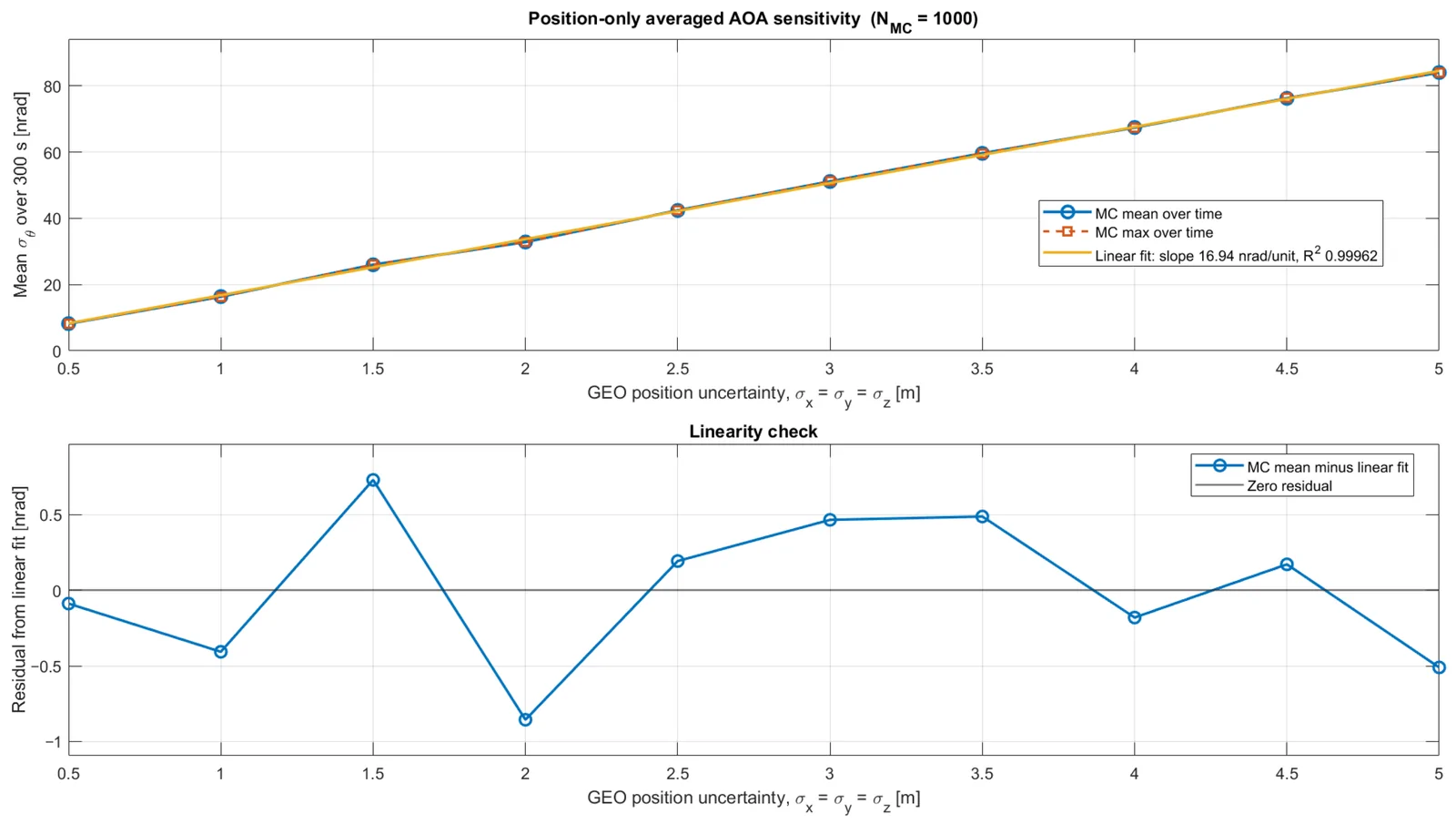
Position-only averaged AOA sensitivity. The (**upper panel**) shows the mean AOA standard deviation over the 300s arc as a function of isotropic, per-axis GEO position uncertainty in the ECI frame. The straight line is the least-squares linear fit, and its slope is the position sensitivity coefficient; the Monte Carlo points and the fit nearly coincide in the (**upper panel**), which is the expected result. The differences are shown in the (**lower panel**), which plots the residual from the linear fit on an expanded scale.

**Figure 10:**
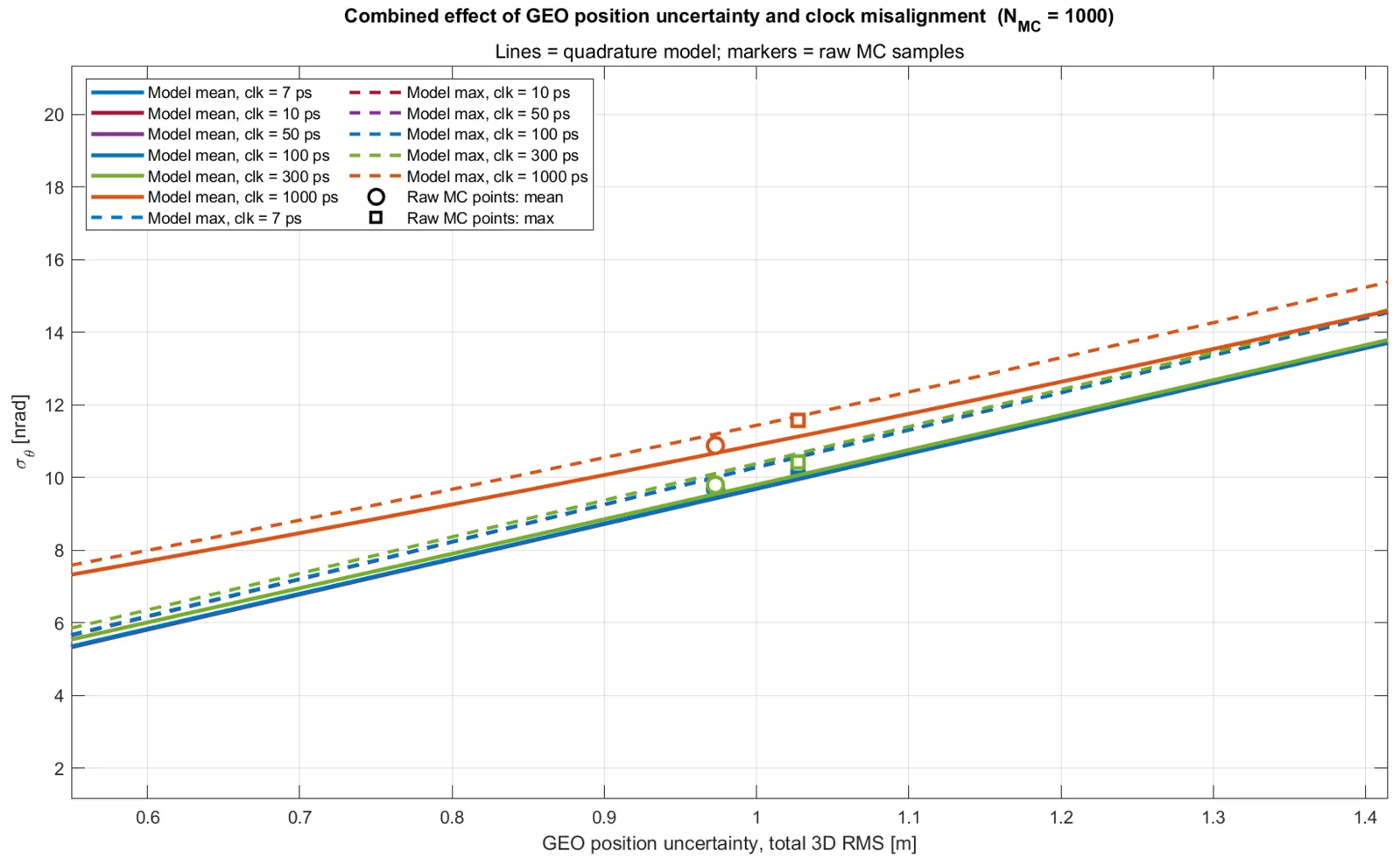
Zoomed-in combined AOA sensitivity at low GEO position uncertainty.

**Table 1:** Allan deviation values for a space-qualified Mini-PHM-class passive hydrogen maser.

Averaging Time τ [s]	Allan Deviation $\sigma_{y}\left(\tau \right)$
1	$6.5\;\times \;10^{-13}$
10	$1.4\;\times \;10^{-13}$
100	$6.3\;\times \;10^{-14}$
1000	$2.2\;\times \;10^{-14}$
10,000	$7.0\;\times \;10^{-15}$
50,000	<1.0 $\times \;10^{-14}$

**Table 2:** Analytical clock contribution to angular uncertainty (in nrad) for a terrestrial-scale baseline and the RINGS trio baseline.

Residual Clock Error	Angular Uncertainty [nrad]	
$\sigma_{t}$ [ps]	Terrestrial Baseline, B=8000km	RINGS Trio Baseline, B=73,000km
10	0.375	0.0411
30	1.12	0.123
100	3.75	0.411
300	11.2	1.23

**Table 3:** Size of the non-reciprocal light-time asymmetry $\Delta \tau_{\mathrm{nr}}$ (Equation (42)), the resulting bias $X=\Delta \tau_{\mathrm{nr}}/2$ of the uncorrected two-way clock-offset estimate, and the Shapiro delay (Equation (40)), for a circular GEO orbit of radius $r_{\mathrm{GEO}}=42,164\;\mathrm{km}$.

Separation α [deg]	Baseline *B* [km]	$\Delta \tau_{\mathrm{nr}}$ [μs]	*X* [μs]	$\Delta t_{\mathrm{Shapiro}}$ [ps]
60	42,164	2.50	1.25	32.5
90	59,629	2.88	1.44	52.2
120	73,030	2.50	1.25	77.9
150	81,455	1.44	0.72	120.0

**Table 4:** Monte Carlo simulation parameters.

Parameter	Value or Range	Basis
Realizations	$N_{\mathrm{MC}}=1000$ per case	Stable estimate of $\sigma_{\theta }$
Observation arc	300s	Coherent window, Section 2.2
Geometry	Circular equatorial GEO, two satellites 180° apart, B≈84,328km, ECI frame	Section 3.1; scaled by 1/B to the trio
Target	$10^{8}\;\mathrm{km}$, RA 45°, Dec 4°, $10\;m\;s^{-1}$	Representative deep-space target
Signal	$f_{1}=3\;\mathrm{GHz}$, $\Delta f_{c}=100\;\mathrm{Hz}$, time step 1s, fixed random seed	Wide-lane observable
Estimator	Dual-frequency wide-lane AOA, Equation (63)	Section 3.3
Clock error	$\delta t_{k}∼N\left(0,\sigma_{t}^{2}\right)$, constant over the arc. Clock-only sweep 5 to 100ps in 5ps steps; cases 7, 10, 50, 100, 300ps; combined sweep 7 to 1000ps	PHM short-term stability (7 to 10ps), conservative residual after calibration (100ps), degraded cases
Position error	$\eta_{r,m}∼N\left(0,\sigma_{\mathrm{ECI}}^{2}I\right)$, independent for the two satellites, constant over the arc. Position-only sweep 0.5 to 5m per axis in 0.5m steps; cases 0.3, 0.5, 1, 2, 5m; combined sweep 0.5 to 5m 3D RMS, redrawn at every epoch	Published precise GEO orbit determination (sub-meter), operational and degraded levels, Section 2.5
Random samples	Common normalized samples across levels in the combined sweep	Reduces draw-to-draw variation

**Table 5:** Representative clock misalignment contribution to RINGS AOA scatter, from Monte Carlo simulation at B≈84,328km.

Clock Misalignment	Mean $\sigma_{\theta }$
$\sigma_{t}$ [ps]	[nrad]
7	0.036
10	0.052
50	0.251
100	0.511
300	1.514

**Table 6:** Combined GEO position and clock sensitivity.

Position RMS	Clock Misalignment	Mean $\sigma_{\theta }$	Max $\sigma_{\theta }$
3D [m]	[ps]	[nrad]	[nrad]
0.5	10	4.85	5.14
0.5	100	4.87	5.18
0.5	1000	6.94	7.29
1.0	10	9.69	10.28
1.0	100	9.70	10.30
1.0	1000	10.88	11.57
2.0	10	19.38	20.56
2.0	100	19.39	20.58
2.0	1000	20.00	21.30
5.0	10	48.45	51.40
5.0	100	48.45	51.41
5.0	1000	48.69	51.78

## Data Availability

The original contributions presented in this study are included in the article. Further inquiries can be directed to the corresponding author.
